# ^68^Ga-Labeled Glycopeptides as Effective Tools for Liver Function Imaging

**DOI:** 10.1021/acs.molpharmaceut.4c01453

**Published:** 2025-02-17

**Authors:** Maximilian Alexander Zierke, Christine Rangger, Kimia Samadikhah, Andreas Martin Schmid, Roland Haubner

**Affiliations:** 1 Department of Nuclear Medicine, Medical University Innsbruck, Anichstr. 35, Innsbruck 6020, Austria; 2 Werner Siemens Imaging Center, Department of Preclinical Imaging and Radiopharmacy, Eberhard Karls University Tübingen, Röntgenweg 13, Tübingen 73076, Germany

**Keywords:** Asialoglycoprotein receptor, functional liver imaging, positron emission tomography, gallium-68, glycopeptides

## Abstract

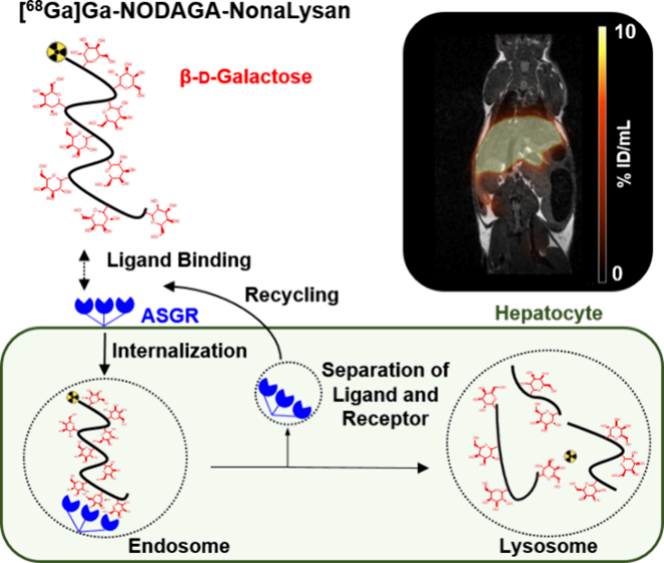

[^99m^Tc]Tc-GSA, an albumin-based glycoprotein, is routinely used in Japan to measure the asialoglycoprotein receptor (ASGR) density via single photon emission tomography. Here we describe the development of ^68^Ga-labeled peptide-based alternatives. Peptides were assembled on a solid support using a fragment coupling strategy. Glycosylation was carried out via a click chemistry approach resulting in a set of three peptides with increasing amounts of d-galactose (*n* = 3, 6, and 9) as well as one glycopeptide bearing nine *N*-acetylgalactosamine residues. ^68^Ga-labeling of all compounds could be achieved in high radiochemical yields (>95%). Radiotracers exhibited high hydrophilicity, good metabolic stability in human serum and protein binding between 12 and 22%. The IC_50_ values improved in the series tri-, hexa-, and nonamer with an IC_50_ of 50 ± 30 pM for the latter one. In analogy, the *in vivo* biodistribution studies revealed increased liver uptake in the series of [^68^Ga]Ga-**NODAGA-TriLysan** (9.4 ± 2.0% ID/g, 30 min p.i.), [^68^Ga]Ga-**NODAGA-HexaLysan** (55.5 ± 7.4% ID/g, 30 min p.i.), and [^68^Ga]Ga-**NODAGA-NonaLysan** (79.6 ± 8.0% ID/g, 30 min p.i.). [^68^Ga]Ga-NODAGA-GalNAc-NonaLysan reached comparable liver uptake to [^68^Ga]Ga-**NODAGA-NonaLysan**, but showed higher accumulation in nontarget organs. The impressive imaging properties of [^68^Ga]Ga-**NODAGA-NonaLysan** were also confirmed by the PET/MR imaging studies in mice. Hence, [^68^Ga]Ga-**NODAGA-NonaLysan** represents a new PET radiopharmaceutical with even better imaging properties than [^99m^Tc]Tc-GSA.

## Introduction

Functional liver imaging addressing the asialoglycoprotein receptor (ASGR) status has gained increased value for disease monitoring because ASGR expression correlates inversely with the progression of various liver-specific diseases such as steatotic liver disease, cirrhosis, and hepatocarcinoma.^1−3^ Furthermore, preoperative assessment of remnant liver function is a key factor for patient outcomes in various clinical settings including surgery and transplantation.^4,5^ The ASGR is a hepatic transmembrane receptor who physiologically clears desialylated glycoproteins from the bloodstream.^6,7^ The receptor is a multimer comprising two H1 and one H2 subunit each bearing a carbohydrate recognition domain with a high affinity for terminal galactose and *N*-acetylgalactosamine residues.^8^ [^99m^Tc]Tc-GSA, a galactosylated human serum albumin-based radiopharmaceutical, targets this receptor and allows the determination of the functional liver mass via SPECT.^9^ Due to its advantage over conventional liver function tests such as the Indocyanine green clearance test or CT-based volumetry, [^99m^Tc]Tc-GSA has found its way into clinical routine in Japan.^3,10^ In Taiwan a clinical phase II study with a compound termed *Dolacga* has been carried out recently. *Dolacga* is the ^68^Ga-labeled version of a dendritic, poly lysine-based galactose hexamer originally termed Hexavalent Lactoside (HexaLac).^11^ It exhibited a low nanomolar binding affinity for the ASGR and showed excellent liver accumulation. Since its first appearance in 2011, several publications have featured the HexaLac structure and evaluated its ASGR targeting properties with different nuclides and chelators including ^111^In-DTPA,^11^^68^Ga-NOTA^12^ and ^18^F[AlF]-NOTA.^13^ Its specificity and accuracy for estimation of the functional liver reserve have further been demonstrated in animal disease models.^14,15^ At least in Europe, the usage of lidocaine analogs such as Mebrofenin is more common.^16−18^ In contrast to the ASGR-targeting radiopharmaceuticals, Mebrofenin binds primarily to albumin, which serves as a shuttle to the liver. There it gets taken up by organic anion-transporting polypeptides (OATP).^19^ Although both tracer classes are suitable for quantification of the functional liver mass, imaging with Mebrofenin comes with several disadvantages. First, Mebrofenin does not get trapped inside the hepatocyte but follows immediate elimination into the bile ducts, thus making it more of an excretion marker.^18^ Second, hypoalbuminemia reduces its delivery to the liver, while renal excretion is increased. Therefore, Mebrofenin uptake can be limited.^20^ Third, high blood bilirubin levels can also reduce hepatic uptake of the tracer as bilirubin and Mebrofenin are both substrates for the OATP.^18^ Unfortunately, a true head-to-head comparison of [^99m^Tc]Tc-GSA and Mebrofenin has not been published yet.

To pave the way for such studies in the future, suitable ASGR tracers would need to be more widely available. So far, our group studied mainly trimeric low molecular weight glycoconjugates with either TRIS^21^ or TRAP^22^ as the central branching unit. However, also peptide-based ligands have been reported as efficient ASGR targeting tools. Examples of this tracer class have been featured in a current review on the research progress of ASGR-targeted radiopharmaceuticals.^23^ As most peptidic ligands bear more than 3 saccharide units we implemented this strategy in our latest synthetic approach. In this article, we now present synthesis and preclinical evaluation of ^68^Ga-labeled glycopeptides for assessment of the functional hepatic reserve with either three, six, or nine saccharide moieties. ^68^Ga was selected as the radionuclide of choice as it comes with a fast and efficient labeling chemistry and good availability from commercial generators.^24^

## Materials and Methods

Solvents, chemicals, and reagents for solid phase peptide synthesis were obtained from Merck (Darmstadt, Germany) or VWR International GmbH (Wien, Austria). 1-Hydroxy-7-azabenzotriazol was purchased from Activate Scientific (Prien am Chiemsee, Germany) as a 1 M solution in DMA. 1-Azido-1-deoxy-β-d-galactopyranoside tetraacetate was obtained from Merck. NODAGA-NHS was purchased from Chematech (Dijon, France). Human recombinant ASGR1 (#4394-AS) was obtained from R&D Systems (Minneapolis, USA). Human α_1_-glycoprotein was commercially available at Merck.

The ^68^Ge/^68^Ga generator, devices for analytical HPLC, semipreparative HPLC, mass spectrometry, radio-thin layer chromatography, gamma counter and animal models for biodistribution and imaging studies including the image reconstruction procedures were the same as described before.^22^ Stability assays in PBS and human blood serum, protein binding as well as determination of log *D* values followed established protocols.^25^ Binding affinities (IC_50_) of selected ligands were determined in an isolated receptor-based assay according to a previously published procedure with minor modifications.^22^ Dilutions of nonradioactive complexes were made with PBS + 0.1% BSA (10^–5^–10^–12^ M). Preparation of [^125^I]I-Asialoorosomucoid which serves as the radioactive standard ligand has been published recently.^21^

Animal experiments were performed in accordance with the Austrian animal experiments law (BGBl. I Nr. 114/2012) and the institution’s animal welfare standards as approved by the Austrian Federal Ministry of Education, Science and Research (BMBWF, 2022–0.311.708). Imaging experiments were conducted at Werner Siemens Imaging Center (Tübingen, Germany) according to the German animal welfare act as approved by the local authorities (R5/19 G, R09/21 G).

Detailed information on the synthesis of the labeling precursor **NODAGA-TriLysan**, **NODAGA-HexaLysan**, **NODAGA-NonaLysan**, and **NODAGA-GalNAc-NonaLysan** as well as mass spectra, and HPLC chromatograms for all compounds can be found in the Supporting Information.

### Radiolabeling

Labeling with ^68^Ga followed a previously published procedure.^26^ In brief, 5 nmol (5 μL, 1 mM) of precursor was mixed with 100 μL of a 1 M NaOAc/HOAc-buffer (pH 5), and 550 μL of ^68^Ga-eluate (approximately 80–100 MBq) was added. The resulting mixture of pH 4.0 was incubated for 15 min at 95 °C at a shaker speed of 1300 rpm. The course of the labeling reaction was monitored on an established radio-TLC and radio-HPLC setup.^22^ [^68^Ga]Ga-**NODAGA-TriLysan** and [^68^Ga]Ga-**NODAGA-HexaLysan** required further purification on a SepPak C_18_ cartridge for biodistribution studies.

### ^nat^Ga-Complexation

The complexation of nonradioactive gallium (^nat^Ga) was achieved by mixing 200 μL of a stock solution (200 nmol, 1 mM) in water/ethanol (1:1; vol/vol) with 6 μL (600 nmol, 100 mM) of a ^nat^GaBr_3_ solution. After 30 min at 75 °C, the nonradioactive complexes were characterized by analytical HPLC and MS.

#### ^nat^Ga-**NODAGA-TriLysan**

RP-HPLC (5–25% B in 15 min): *t*_R_ = 10.6 min (18% B);

calculated monoisotopic mass (C_88_H_141_GaN_26_O_35_): 2190.93 Da;

found (*m*/*z*) = 1097.3 [M + 2H]^2+^, 732.02 [M + 3H]^3+^, 474.1 [M + 1H + 4K]^5+^.

#### ^nat^Ga-**NODAGA-HexaLysan**

RP-HPLC (5–25% B in 15 min): *t*_R_ = 11.5 min (19% B); calculated monoisotopic mass (C_156_H_249_GaN_47_O_61_): 3856.73 Da;

found (*m*/*z*) = 1294.4 [M + 2H + Na]^3+^, 1286.6 [M + 3H]^3+^, 965.4 [M + 4H]^4+^, 772.5 [M + 5H]^5+^,

564.8 [M + 3H + 4Na]^7+^.

#### ^nat^Ga-**NODAGA-NonaLysan**

RP-HPLC (5–25% B in 15 min): *t*_R_ = 12.0 min (20% B); calculated monoisotopic mass (C_226_H_363_GaN_68_O_89_): 5522.52 Da; found (*m*/*z*) = 1840.5 [M + 3H]^3+^, 1381.4 [M + 4H]^4+^, 1105.9 [M + 5H]^5+^, 921.6 [M + 6H]^6+^.

582.8 [M + 4Na + 6K]^10+^,

#### ^nat^Ga-**NODAGA-GalNAc-NonaLysan**

RP-HPLC (5–25% B in 15 min): *t*_R_ = 12.8 min (21% B); calculated monoisotopic mass (C_244_H_390_GaN_77_O_89_): 5891.76 Da; found (*m*/*z*) = 1496.8 [M + 4Na]^4+^, 1196.0 [M + 1H + 4Na]^5+^, 1000.2 [M + 1H + 5Na]^6+^.

### Statistical Analysis

The statistical significance of experimental data was calculated in SPSS using a student’s *t-*test for unpaired samples.

## Results

### Chemical Synthesis

**Scheme 1:**
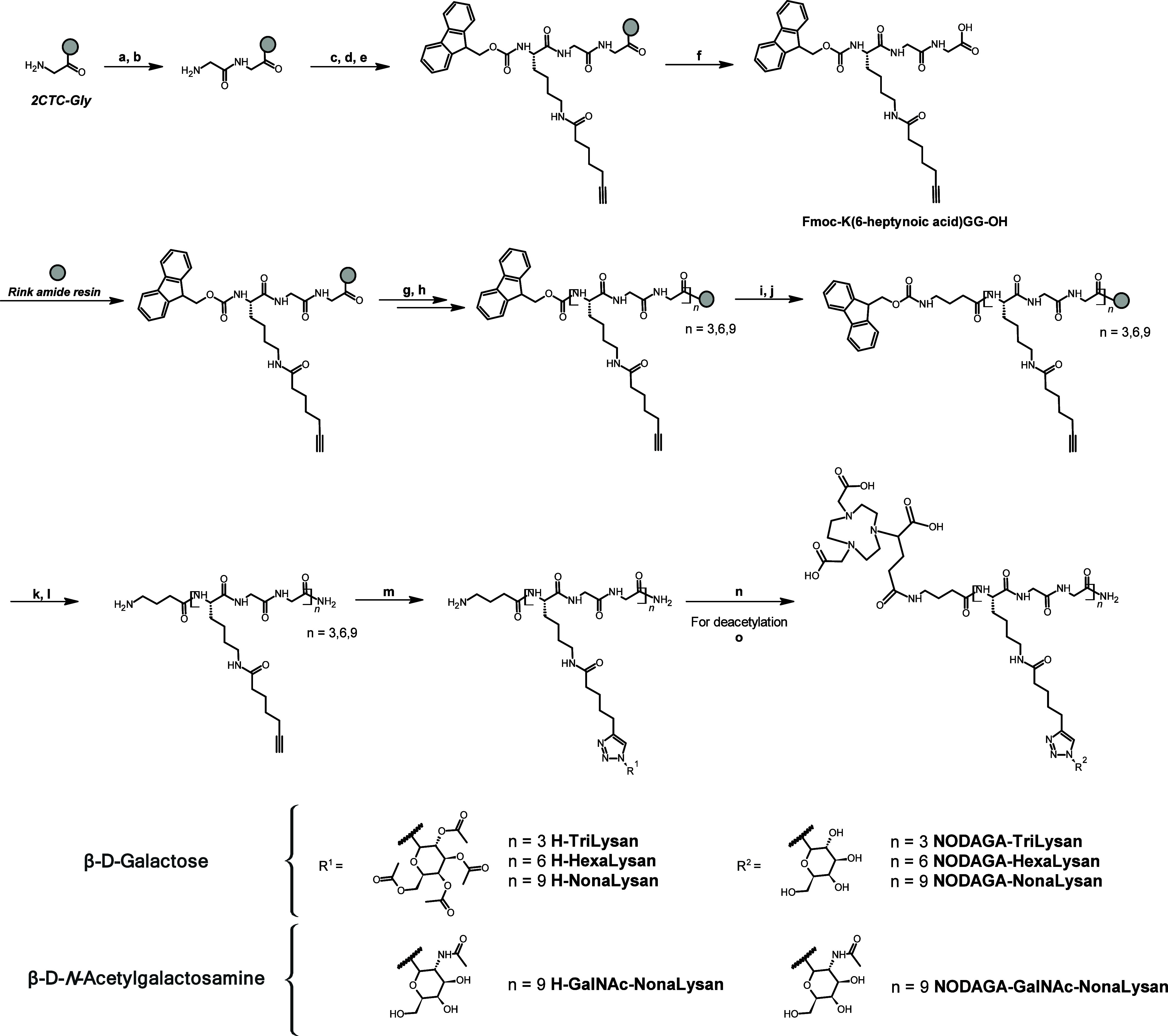
Synthetic Route of Glycopeptides by a Fragment Condensation Approach. First, Fmoc-K(6-heptynoic acid)GG-OH Is Prepared on a CTC Resin. Second, The Preassembled Tripeptide Gets Immobilized on a Rink Amide Resin for Repetitive Coupling Cycles. Conjugation of the Glycosides and the Chelator Were Performed in Solution after Cleavage from the Solid Support Fmoc-Gly, HOAt, HATU, DIPEA (DMF). Pip/DMF (20%). Fmoc-Lys(Dde)-OH, HOAt, HATU, DIPEA (DMF). NH_3_OHCl, Imidazole (NMP/DMF). 6-heptynoic acid, HOAt, HATU, DIPEA (DMF). TFA/TIPS/H_2_O; yield: 87%. Pip/DMF (20%). Fmoc-K(6-heptynoic acid)GG-OH, HOAt, HATU, DIPEA. Pip/DMF (20%). Fmoc-GABA–OH, HOAt, HATU, DIPEA (DMF). Pip/DMF (20%). TFA/TIPS/H_2_O; yield: 10–56%. β-glycosyl azide, Cu(OAc)_2_*H_2_O, sodium ascorbate (*t*BuOH/H_2_O); yield: 13–45%. NODAGA-NHS, DIPEA (DMSO). NEt_3_/MeOH/H_2_O; yield: 21–91%.

The synthesis of all labeling precursors was achieved via fragment condensation of the preassembled Lys-Gly-Gly (KGG) amino acid motif on a Rink amide resin using Fmoc-chemistry (Scheme 1). Therefore, peptides with either three, six, or nine lysine derivatives could be obtained. Finally, the *N*-terminus was elongated with Fmoc-γ-butyric acid and after a last deprotection step, the peptide was cleaved from the solid support. The acetyl-protected β-glycosyl azides were attached to the peptidic backbone via copper-catalyzed azide–alkyne cycloaddition. In the last step, the free amino function on the *N*-terminus was reacted with NODAGA-NHS-ester. In the case of galactose, peptides were treated with a mixture of triethylamine/water/methanol for subsequent deacetylation. Following semipreparative HPLC purification, the glycopeptides were obtained in yields ranging from 1 to 3% and high purities (93–97%) (Table 1).

**Table 1:** MS Data, HPLC Retention Time, Purity, and Yields of the Glycopeptides

	**monoisotopic mass calcd. (Da)**	**monoisotopic mass found**(*m*/*z*)	*t*_R_**HPLC** (min)^a^	**purity** (%)^b^	**yield** (%)^c^
**NODAGA-TriLysan**	2125.03	2147.2 [M + Na]^+^	11.0	>97	3
**NODAGA-HexaLysan**	3790.82	3793.82 [M + H]^+^	11.8	>93	1
**NODAGA-NonaLysan**	5456.62	1820.88 [M+3H]^3+^	12.3	>97	2
**NODAGA-GalNAc-NonaLysan**	5825.86	5826.85 [M + H]^+^	12.9	>96	1

a Gradient: 5–25% B in 15 min, 1 mL/min.

b Determined by HPLC.

c Calculated based on the initial amount of Fmoc-K(6-heptynoic acid)GG-OH immobilized on the Rink amide resin.

### Gallium-Labeling Procedures

**Figure 1:**
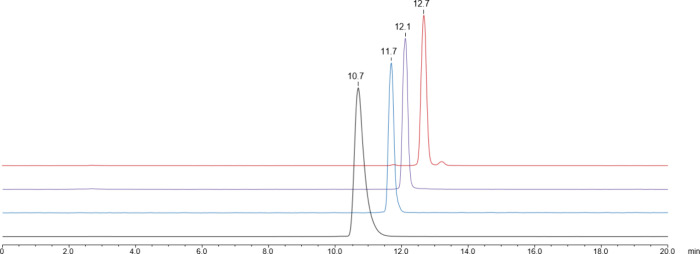
Radio-HPLC chromatograms of [^68^Ga]Ga-**NODAGA-TriLysan** (black), [^68^Ga]Ga-**NODAGA-HexaLysan** (blue), [^68^Ga]Ga-**NODAGA-NonaLysan** (violet), and [^68^Ga]Ga-**NODAGA-GalNAc-NonaLysan** (red). Column: Dr. Maisch ReproSil Pur C_18_ AQ, 150 × 4.6 mm, 5 μm, 120 Å; Solvent A: H_2_O/0.1% TFA, Solvent B: MeCN/0.1% TFA; Flow: 1 mL/min; Gradient: 5–25% B in 15 min.

All peptides could be labeled with ^68^Ga within 15 min at 95 °C and were obtained in high (>95%) radiochemical purity. However, a complete ^68^Ga-incorporation (≥99% RCY) occurred only with the nonameric compounds. Molar activities ranged between 18 and 24 MBq/nmol. Radio-HPLC chromatograms showed that retention times increase with the molecular weight of the complexes (Figure 1).

### *InVitro* Evaluation

All compounds showed high stability in human blood serum (*n* = 2) and PBS (*n* = 1) (Table 2). Protein binding was low to moderate with [^68^Ga]Ga-**NODAGA-TriLysan** and [^68^Ga]Ga-**NODAGA-GalNAc-NonaLysan** showing the least interaction with serum proteins after 120 min of incubation (13.6 ± 3.1% (*n* = 2) and 11.6 ± 2.4% (*n* = 2)). In contrast, the highest amount of protein binding was found for [^68^Ga]Ga-**NODAGA-NonaLysan** and [^68^Ga]Ga-**NODAGA-HexaLysan** (18.7 ± 1.9% (*n* = 2) and 21.5 ± 3.4% (*n* = 2), 120 min). All peptides exhibited high hydrophilicity with log *D* values ranging from −3.52 ± 0.03 (*n* = 8) to −4.34 ± 0.13 (*n* = 5). IC_50_-determinations in an isolated receptor-based assay revealed that binding affinity toward the ASGR shifts from nanomolar to picomolar when the amount of galactose exceeds the number of three as shown for ^nat^Ga-**NODAGA-HexaLysan** and ^nat^Ga-**NODAGA-NonaLysan**.

**Table 2:** Serum and PBS Stability, Protein Binding, IC_50_, and log *D* of ^68^Ga-Labeled Glycopeptides

	**complex stability in serum** (% intact ligand)				**complex stability in PBS** (% intact ligand)				**protein binding** (%)				**IC**_**50**_ (nM)^a^	**log *D***
**min**	**2**	**30**	**60**	**120**	**2**	**30**	**60**	**120**	**2**	**30**	**60**	**120**		
[^68^Ga]Ga-**NODAGA-TriLysan**	99.9	99.9	99.9	99.9	99.7	99.8	99.8	99.8	7.6 ± 3.9	5.5 ± 0.5	9.2 ± 0.2	13.6 ± 3.1	15.2 ± 8.0	–3.52 ± 0.03
[^68^Ga]Ga-**NODAGA-HexaLysan**	99.4 ± 0.2	99.7 ± 0.1	99.9 ± 0.1	99.9 ± 0.1	99.9	99.9	99.9	99.9	17.6 ± 2.2	21.8 ± 4.4	22.0 ± 2.5	18.7 ± 1.9	0.4 ± 0.4	–4.34 ± 0.13
[^68^Ga]Ga-**NODAGA-NonaLysan**	99.5 ± 0.1	99.5 ± 0.1	99.5 ± 0.1	99.6	99.6	99.4	99.3	99.3	15.3 ± 4.2	28.3 ± 2.9	26.3 ± 1.0	21.5 ± 3.4	0.05 ± 0.03	–4.06 ± 0.09
[^68^Ga]Ga-**NODAGA-GalNAc-NonaLysan**	98.8 ± 0.1	98.8	99.0 ± 0.2	98.8 ± 0.2	99.1	99.0	99.1	99.1	14.2 ± 5.6	14.0 ± 5.7	17.4 ± 3.5	11.6 ± 2.4	n.d.^b^	–4.05 ± 0.03

a Half-maximum inhibitory concentration of [^125^I]I-Asialoorosomucoid bound to human recombinant ASGR1; for this assay, the nonradioactive gallium complexes were used.

b Not detemined.

### *In Vivo* Evaluation

**Figure 2:**
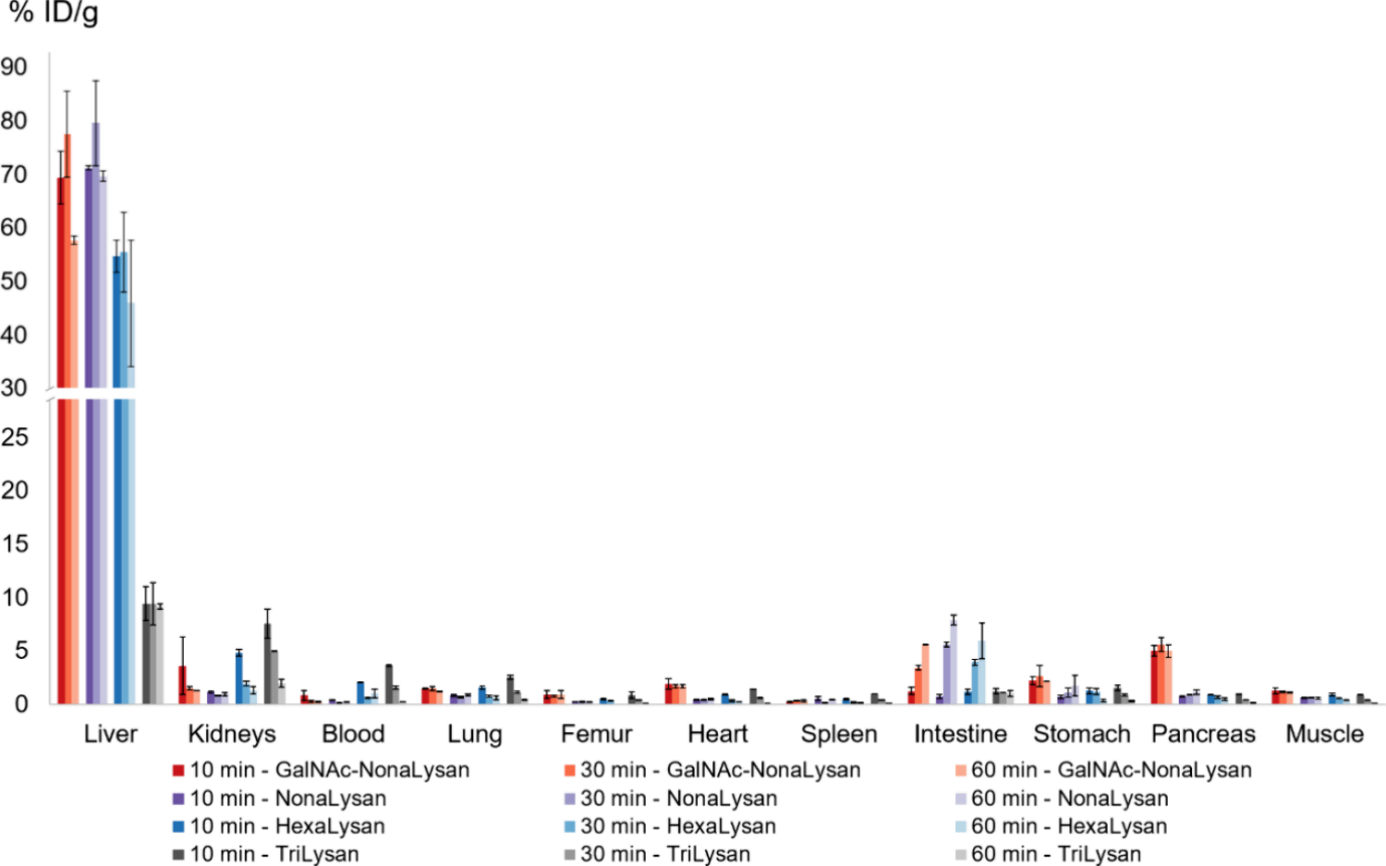
Biodistribution data (% ID/g) of ^68^Ga-labeled **NODAGA-GalNAc-NonaLysan**, **NODAGA-NonaLysan**, **NODAGA-HexaLysan**, and **NODAGA-TriLysan** in healthy BALB/c mice at 10, 30, and 60 min p.i. (100 pmol, 1 MBq).

**Figure 3:**
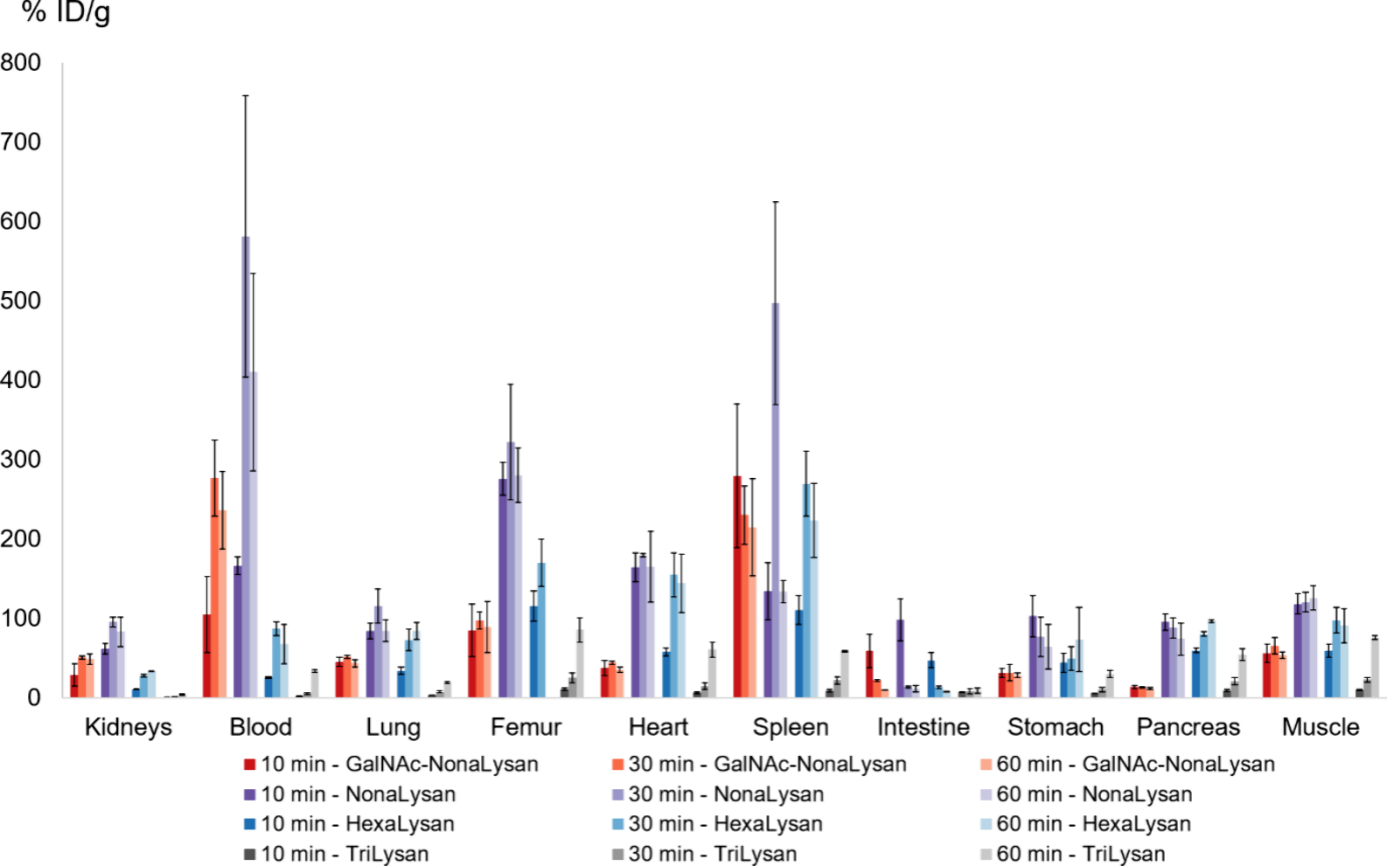
Liver-to-organ ratios of ^68^Ga-labeled **NODAGA-GalNAc-NonaLysan**, **NODAGA-NonaLysan**, **NODAGA-HexaLysan**, and **NODAGA-TriLysan** in healthy BALB/c mice at 10, 30, and 60 min p.i. (100 pmol, 1 MBq).

**Figure 4:**
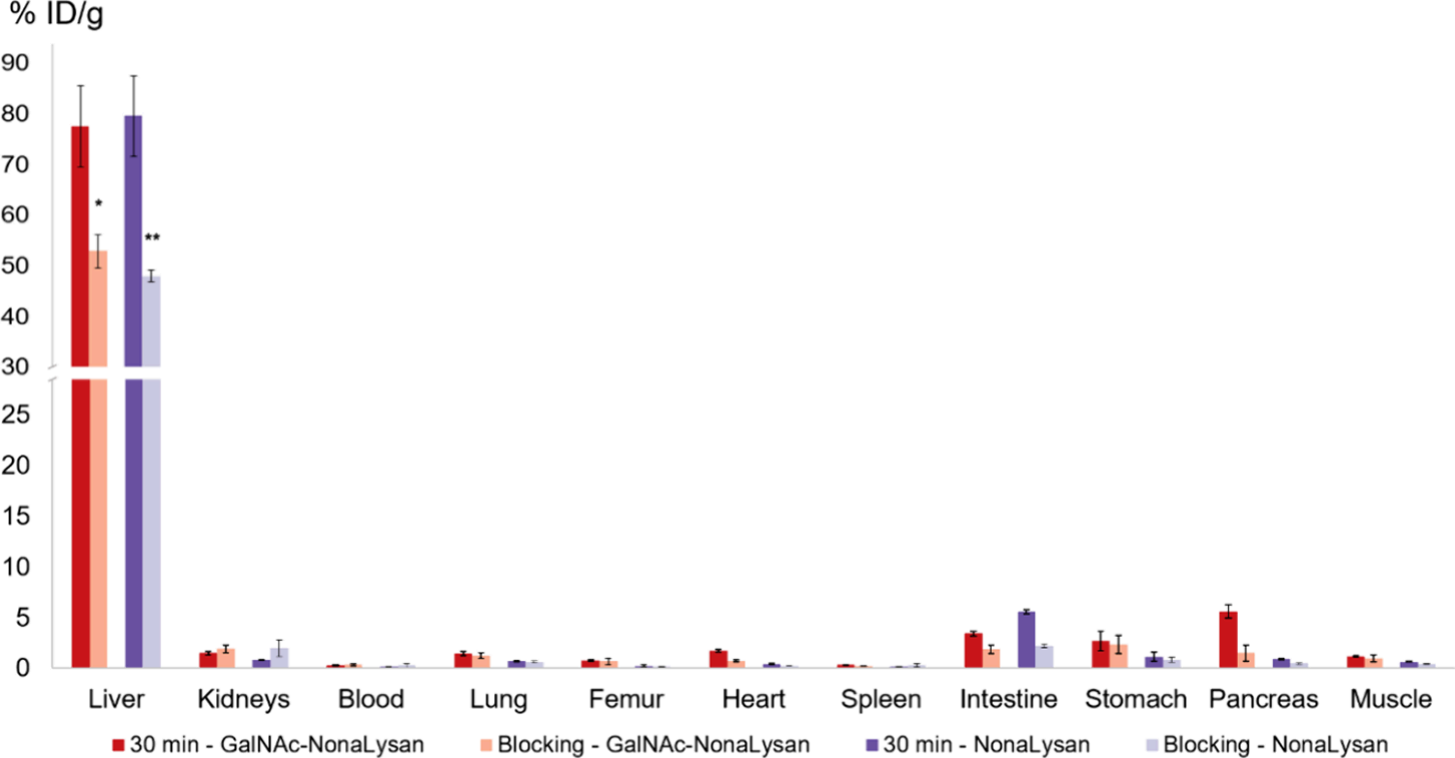
Data (% ID/g) from the blocking experiments using ^68^Ga-labeled **NODAGA-GalNAc-NonaLysan** and **NODAGA-NonaLysan** in healthy BALB/c mice at 30 min p.i. Blocking was carried out using 27.7 μmol of *N*-acetylgalactosamine (**p ≤ 0.01; *p ≤ 0.05).

Biodistribution studies in healthy female BALB/c mice revealed the highest liver uptake at 30 min p.i. for [^68^Ga]Ga-**NODAGA-NonaLysan** (79.6 ± 8.0% ID/g) and its twin [^68^Ga]Ga-**NODAGA-GalNAc-Nonalysan** (77.6 ± 8.0% ID/g) (Figure 2 and Tables S1 and S2). Less liver uptake was found for [^68^Ga]Ga-**NODAGA-HexaLysan** (55.5 ± 7.4% ID/g, 30 min p.i.) and least for [^68^Ga]Ga-**NODAGA-TriLysan** (9.4 ± 2.0% ID/g, 30 min p.i.). All compounds showed low blood level activity and only minor accumulation in nontarget tissue. However, an increasing activity accumulation in the intestine was found for [^68^Ga]Ga-**NODAGA-NonaLysan**, [^68^Ga]Ga-**NODAGA-GalNAc-Nonalysan,** and [^68^Ga]Ga-**NODAGA-HexaLysan** over time. Nevertheless, liver-to-organ ratios were highest for [^68^Ga]Ga-**NODAGA-NonaLysan** (Figure 3). Lower liver-to-organ ratios were found for the GalNAc derivative due to higher off-target binding in the pancreas, stomach, heart, muscle, lung, and femur. By far the lowest contrast was found for [^68^Ga]Ga-**NODAGA-TriLysan** due to its overall weak liver accumulation and elevated kidney uptake, probably caused by renal excretion. Coinjection of both nonamers with a large excess of *N*-acetylgalactosamine led to a statistically significant reduction of liver uptake indicating specific interactions of the tracers with their target (Figure 4). As a consequence of the reduced liver uptake also a reduction of the intestinal uptake was observed.

### *InVivo* Imaging

**Figure 5:**
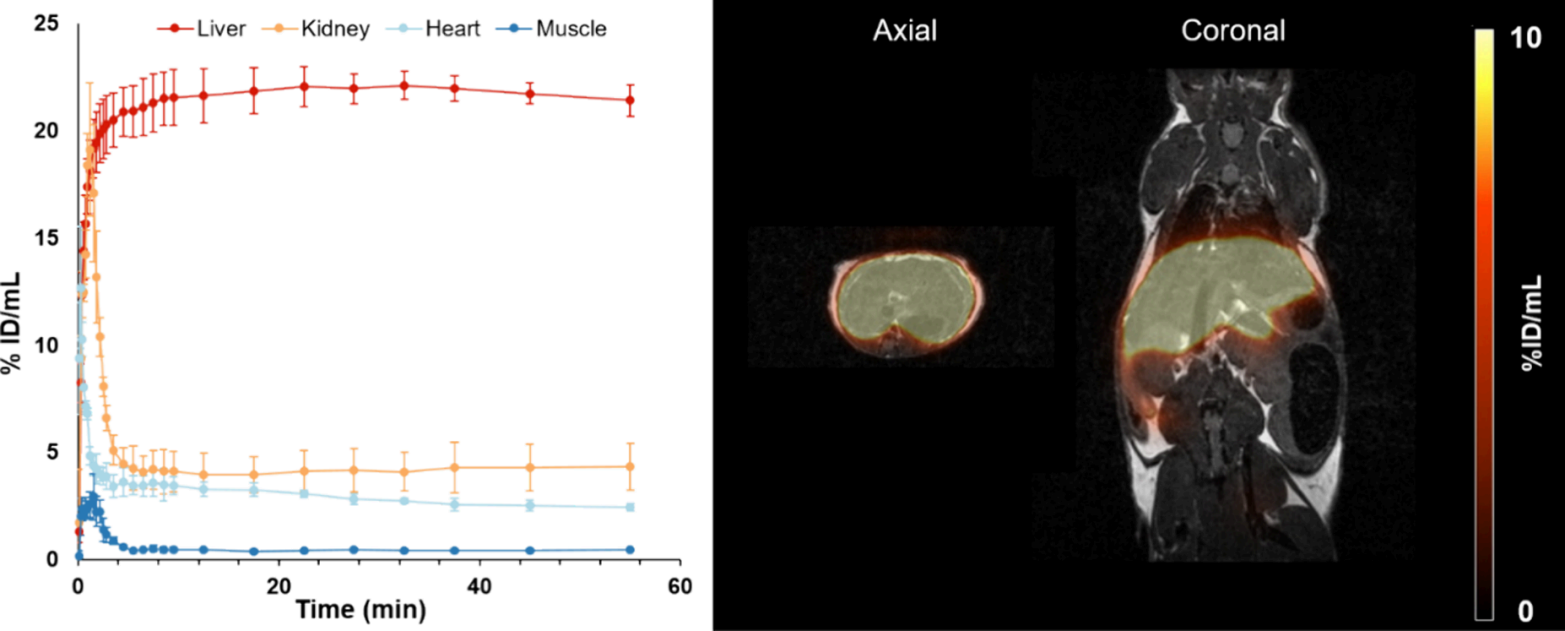
TACS of liver, kidney, heart, and muscle of the imaged mice (*n* = 3; left) and a representative PET/MR fusion image (right) of one healthy male C57BL6 mouse, injected with 1 MBq (100 pmol) of [^68^Ga]Ga-**NODAGA-NonaLysan**.

The best glycopeptide from biodistribution studies was further evaluated with PET/MR in a C57BL6 mouse model (Figure 5). The pharmacokinetic profile of [^68^Ga]Ga-**NODAGA-NonaLysan** showed a fast liver accumulation within the first 2 min of the observation period followed by a stable retention of the activity over rest of the scan. Quick washout from all nontarget tissue resulted in a high-contrast image allowing a precise delineation of the functional hepatic reserve. Quantification of the hepatic uptake by drawing regions of interest over liver, kidneys, muscle, and heart demonstrated an excellent separation of the time activity curves.

## Discussion

Until today [^99m^Tc]Tc-GSA is the “gold standard” compound for assessment of the functional liver mass in cases of acute liver injury, surgery, transplantation, and radiation therapy of tumors. However, the fact that this radiopharmaceutical is commercially distributed only in Japan makes it largely inaccessible for Western nuclear medicine departments.^27^ Attempts to make this compound available for PET imaging resulted in the generation of [^68^Ga]Ga-NOTA-GSA with similar imaging properties compared to [^68^Ga]Ga-DTPA-GSA, but increased serum stability.^28^ Despite the intriguing preclinical data, further clinical translation failed due to the unavailability of a GMP-compliant labeling precursor based on human serum albumin. Consequently our research has focused on the development of small molecule-based radiotracers, lately, as these compounds can be more easily produced under GMP-compliant methods.

Our latest synthetic approach is based on a single linear amino acid sequence containing different amounts of glycosylated lysines with two glycines as spacers in between. These comb-like structures exhibited high stability and comparable hydrophilicity among each other. Binding affinities for the isolated ASGR1 increased in the order of trimer < hexamer < nonamer from the low nanomolar to the picomolar range for the galactosylated peptides. This phenomenon has already been reported in the literature as the so-called “cluster effect” and describes the preference of the ASGR for multimerized ligands.^29^ However, it is important to know that also the geometry of the presented galactose moieties plays an important role in high-affinity interactions with the receptor. According to structure–activity studies, trimers are most effective if they bear the galactose residues at a specific distance of 18–20 Å.^30,31^ The very low liver accumulation of [^68^Ga]Ga-**NODAGA-TriLysan** might be explained by the unfavorable presentation of the 3 galactose units in this compound (Figure 2). In contrast to symmetric trimers based on TRIS or TRAP, here the galactose residues are attached to a linear peptide sequence. Having the receptor geometry in mind it is rather unlikely that these 3 galactose residues align in a perfect triangular geometry as required for high affinity interactions. However, the targeting properties of these linear compounds improve remarkably upon multiplying the amount of binding motives to 6 or 9 galactose units. As a result, the liver uptake found for [^68^Ga]Ga-**NODAGA-HexaLysan** (55.7 ± 7.4% ID/g, 30 min p.i.) is already comparable to [^99m^Tc]Tc-GSA (51.3 ± 2.8% ID/g, 30 min p.i.), while [^68^Ga]Ga-**NODAGA-NonaLysan** shows statistically significant higher liver accumulation than the Japanese reference compound (*p* ≤ 0.01 at 30 min.p.i.; *p* ≤ 0.05 at 60 min p.i.).^21^ Unfortunately, no biodistribution data have been reported for *Dolacga*, but the data set for [^111^In]In-HexaLac indicates that after 10 min p.i. about 72.6 ± 4.6% ID/g can be found in the liver and at 60 min p.i. it decreases to about 60% ID/g.^14^ Similar values were obtained for [^68^Ga]Ga-**NODAGA-NonaLysan**, ranging between 71.2 ± 0.4% ID/g and 69.6 ± 1.0% ID/g at 10 and 60 min p.i. The impressive imaging characteristics of [^99m^Tc]Tc-GSA and [^111^In]In-HexaLac are further enhanced by their negligible off-target binding. In the case of [^68^Ga]Ga-**NODAGA-NonaLysan** the highest nontarget uptake is found within the intestine, which might be due to partial hepatobiliary excretion of the tracer. However, with a maximum value of 7.9 ± 0.5% ID/g 60 min p.i. it is still lower than the intestinal activity found for [^99m^Tc]Tc-GSA (9.8 ± 1.9% ID/g 60 min p.i.).^21^

Specificity for the interaction of [^111^In]-HexaLac with the ASGR was demonstrated by a blocking experiment. Upon coinjection of 100 μg asialofetuin liver uptake could be reduced to a value as low as 0.41 ± 0.04% ID/g. In the case of [^68^Ga]Ga-**NODAGA-NonaLysan**, 27.7 μmol GalNAc was used as the blocking agent of choice. *N*-acetylgalactosamine has been described as an efficient blocking substance for *in vitro* assays.^32−34^ Indeed, a statistically significant reduction in liver uptake could be detected. But unfortunately, the blocking was not as efficient as reported for [^111^In]In-HexaLac. This might be because the monomeric carbohydrate is not the optimal blocking agent for such a high-affinity probe.

The imaging data of [^68^Ga]Ga-NOTA-GSA, *Dolacga,* and [^68^Ga]Ga-**NODAGA-NonaLysan** allow a clear delineation of the functional liver mass. Time-activity curves of the liver show a rapid tracer accumulation within the first 5 min, followed by a stable retention of the activity until the end of the observation period for all tracers.^14,28^ It is assumed that an initial high and stable retention of the activity is beneficial for the image interpretability because the ASGR expression is known to be decreased in diseased hepatic tissue.

Attempts to further optimize our best-performing peptide resulted in the generation of [^68^Ga]Ga-**NODAGA-GalNAc-NonaLysan**. Previous studies have shown that the exchange of galactose by *N*-acetylgalactosamine led to a significant increase in liver uptake, while off-target binding was reduced simultaneously.^22^ In this case, however, the replacement of galactose by *N*-acetylgalactosamine resulted in no further improvement of the radiopharmaceutical as neither tracer uptake in the liver nor elimination was enhanced. Our results indicate that with almost 80% ID/g liver uptake is at a maximum. Instead, increased off-target binding was found for [^68^Ga]Ga-**NODAGA-GalNAc-NonaLysan** in lung, femur, heart, stomach, pancreas, and muscle resulting in lower liver-to-organ ratios. Upon coinjection of 27.7 μmol GalNAc into healthy female BALB/c mice, a statistically significant reduction in liver uptake was registered. But surprisingly, also a reduction in heart and pancreas uptake was found, which was not observed for [^68^Ga]Ga-**NODAGA-NonaLysan**. It is not known why the GalNAc-multimer would accumulate specifically in these organs and at this point, we can only speculate about possible explanations. However, as we opted for [^68^Ga]Ga-**NODAGA-NonaLysan** as the lead compound, further investigations in this regard were omitted.

## Conclusions

In this article, we present the successful synthesis of ^68^Ga-labeled linear glycopeptides and the evaluation of their suitability as imaging agents for the ASGR. Within the tested set [^68^Ga]Ga-**NODAGA-NonaLysan** was the most potent candidate exhibiting subnanomolar ASGR affinity and excellent liver-targeting properties with minimal accumulation in nontarget tissue. The superior pharmacokinetic profile makes peptidic ASGR tracers a valuable alternative to trimeric glycoconjugates. Additionally, peptides can be produced in a GMP-compliant manner and have been reported in combination with various radionuclides, e.g., gallium-68,^35^ fluorine-18,^36^ or indium-111.^37^ Hence, we would propose to conduct subsequent clinical studies with [^68^Ga]Ga-**NODAGA-NonaLysan** shortly.

## Data Availability

The analyzed data sets generated during the present study are available from the corresponding author on reasonable request.

## Supporting Information Available

General procedures for solid phase peptide synthesis; synthesis of Fmoc-K(6-heptynoic acid)GG-OH, characterization with RP-HPLC and MS (Figures S1 and S2); synthesis of **NODAGA-TriLysan**, characterization with RP-HPLC and MS (Figures S3 and S4); synthesis of **NODAGA-HexaLysan**, characterization with RP-HPLC and MS (Figures S5 and S6); synthesis of **NODAGA-NonaLysan**, characterization with RP-HPLC and MS (Figures S7 and S8); synthesis of **H-GalNAc-NonaLysan**, characterization with RP-HPLC and MS (Figures S9 and S10); synthesis of **NODAGA-GalNAc-NonaLysan**, characterization with RP-HPLC and MS (Figures S11 and S12); biodistribution data of [^68^Ga]Ga-**NODAGA-TriLysan**, [^68^Ga]Ga-**NODAGA-HexaLysan**, [^68^Ga]Ga-**NODAGA-NonaLysan**, and [^68^Ga]Ga-**NODAGA-GalNAc-NonaLysan** (Tables S1 and S2) (PDF)

## Abbreviations Used

%ID/g: percent internalized dose per gram
ASGR: asialoglycoprotein receptor
ASOR: asialoorosomucoid
BSA: bovine serum albumin
Calcd: calculated
DIPEA: diisopropylethylamine
DMA: dimethylacetamide
DMF: dimethylformamide
DMSO: dimethyl sulfoxide
GalNAc: *N*-acetylgalactosamine
GSA: galactosylated human serum albumin
HATU: O-7-azabenzotriazol-1-yl-N,N,*N*′,*N*′-tetramethyluronium-hexafluorophosphat
HOAT: 1-hydroxy-7-azabenzotriazole
HPLC: high performance liquid chromatography
KGG: lysine-glycine-glycine
MeCN: acetonitrile
MeOH: methanol
NaOH: sodium hydroxide
NEt_3_: trimethylamine
NMP: *N*-methyl-2-pyrrolidone
OATP: organic anion transporting polypeptides
p.i.: post injection
PBS: phosphate-buffered saline
PET: positron emission tomography
SPECT: single photon emission tomography
TAC: time activity curve
TBS: tris-buffered saline
TFA: trifluoroacetic acid
TIPS: triisopropylsilane
*t*_R_: retention time
TRAP: triazacyclononane phosphinic acid
TRIS: tris(hydroxymethyl)aminomethane
