# Supplementary material for: 68Ga-Labeled Glycopeptides as Effective Tools for Liver Function Imaging
Source: Mol Pharm. 2025 Feb 17;22(3):1677–85. doi: 10.1021/acs.molpharmaceut.4c01453 (PMC11881035; doi:10.1021/acs.molpharmaceut.4c01453)
Supplement: Supplementary file 1 — mp4c01453_si_001.pdf [file mp4c01453_si_001.pdf]

Supporting Information

^{68}Ga-Labeled Glycopeptides as Effective Tools for Liver Function Imaging

Maximilian Alexander Zierke[1], Christine Rangger[1], Kimia Samadikhah[2], Andreas Martin Schmid[2] & Roland Haubner[1,§]

[1]Department of Nuclear Medicine, Medical University Innsbruck, Anichstr. 35, 6020 Innsbruck, Austria

[2]Werner Siemens Imaging Center, Department of Preclinical Imaging and Radiopharmacy, Eberhard Karls University Tübingen, Röntgenweg 13, 73076 Tübingen, Germany

§to whom correspondence should be addressed

Roland Haubner, PhD, Medical University Innsbruck. E-mail: roland.haubner@i-med.ac.at

Table of Content

1. General Procedures for Solid Phase Peptide Chemistry 3

2. Synthetic Procedures and Analytical Data 4

2.1 Fmoc-K(6-heptynoic acid)-GG-OH 4

2.2 NODAGA-TriLysan... 6

2.3 NODAGA-HexaLysan 8

2.4 NODAGA-NonaLysan 10

2.5 H-GalNAc-NonaLysan... 12

2.6 NODAGA-GalNAc-NonaLysan 13

3. Additional Tables... 15

1. General Procedures for Solid Phase Peptide Chemistry

Loading of fragments on to a Rink amide resin (GP1)

Rink amide resin (80 mg, δ = 0.65 mmol/g, 1.0 eq) was weighed into a 10 mL syringe equipped with a pp-frit inlet and was swollen in DMF for 1 hour. Removal of the first Fmoc-protection group was accomplished by incubating the resin for 1 hour with a mixture of 20 % Pip/DMF (vol/vol). The resin was washed with DMF (8 × 3 mL) and a solution of 0.9 eq Fmoc-K(6-heptynoic acid)GG-OH, 0.9 eq HOAt, 0.9 eq HATU and 4.5 eq DIPEA in DMF was added. After 24 hours of incubation time the resin was washed with DMF (3 × 3 mL) and all non-reacted active sites were capped by adding a mixture of Acetic Anhydride/Pyridine (3:2) (vol/vol) for 30 min. The resin was washed once again with DMF (3 × 3 mL) and was ready to use for the next step.

On-resin peptide bond formation for single amino acids (GP2a)

In a 5 mL glass vial HOAt (2.0 eq), HATU (2.0 eq) and the respective amino acid (2.0 eq) were dissolved in 3 mL of DMF with stirring. DIPEA (6.0 eq) was added and the solution was stirred for another 5 min at room temperature. The mixture was added to the resin and the reaction was allowed to continue for 2 hours.

Fragment Coupling (GP2b)

Fmoc-K(6-heptynoic acid)GG-OH (1.0 eq) was weighed into a 5 mL glass vial together with 1.0 eq HOAt and 1.0 eq HATU. DMF (2 mL) and DIPEA (6.0 eq) were added and the solution was stirred for 5 min at room temperature. This mixture was then added to the resin and the reaction was allowed to continue for 3 hours.

Fmoc-Removal (GP3)

The resin was treated with a mixture of 20 % Pip/DMF (vol/vol) (1 × 5 min, 1 × 15 min) and was washed with DMF afterwards. (8 × 6 mL/g resin).

On-resin Dde-deprotection (*GP4*)

For on resin Dde-deprotection in the presence of Fmoc-groups the resin was treated with a solution of Imidazole (0.92 g/g resin) and Hydroxylamin Hydrochloride (1.26 g/g resin) in 5 mL of NMP and 1 mL of DMF. The solution was added to the resin for a time period of 3 hours and the resin was washed with DMF (3 × 6 mL/g resin) afterwards.

Capping of unreacted amines (GP5)

The resin was treated for 30 min with 5 mL of a freshly prepared Acetic Anhydride/Pyridine (3:2) solution. Afterwards the resin was washed 3 times with DMF.

Cleavage of peptides from the resin (GP6)

The resin was washed thoroughly with DCM (3 × 6 mL) before 3 mL of a cleavage cocktail containing TFA/TIPS/H_2O (95/2.5/2.5) (vol/vol/vol) were added. After 45 min the solution containing

the crude peptide was transferred into a 15 mL round bottom flask and 2 mL of new cleavage cocktail were added to the syringe. This step was repeated three times in total. The combined fractions were reduced under a stream of Argon and either precipitated in ice-cold diethylether or directly lyophylized in a mixture of H_2O/*tBu*OH (1:1) (vol/vol).

2. Synthetic Procedures and Analytical Data

2.1 Fmoc-K(6-heptynoic acid)-GG-OH

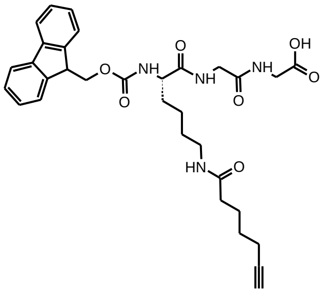

Fmoc-K(6-heptynoic acid)-GG-OH

Mw: 590.67 g/mol
Monoisotopic Mass: 590.27 Da
$C_{32}H_{38}N_4O_7$

Assembly of the alkyne tagged KGG-motif, which serves as the general building block, was achieved via solid phase peptide synthesis starting with 500 mg (550 µmol) of glycine-loaded *CTC*-resin (δ = 1.10 mmol/g). Coupling of glycine and Dde-protected L-lysine was performed accordingly to a general procedure *(GP2a & GP3)*. On-resin Dde-removal was carried out using Imidazole and Hydroxylammoniumchloride *(GP4)*. Attachment of 6-heptynoic acid to the lysine sidechain was accomplished using HOAt (1.2 eq), HATU (1.2 eq) and DIPEA (3.0 eq) over the course of 3.5 hours. Cleavage of the motif from the resin was done according to *GP6*. Purification via semi-preparative HPLC (43-55 % B in 25 min) and subsequent lyophylization yielded 316 mg (536 µmol, 87 %) of a colorless solid.

RP-HPLC (20-80 % B in 15 min) t_R = 9.0 min (52 % B).

MALDI-MS (m/z) = 591.5 $[M+H]^+$, 613.5 $[M+Na]^+$, 629.5 $[M+K]^+$, 651.4 $[M+Na+K]^+$.

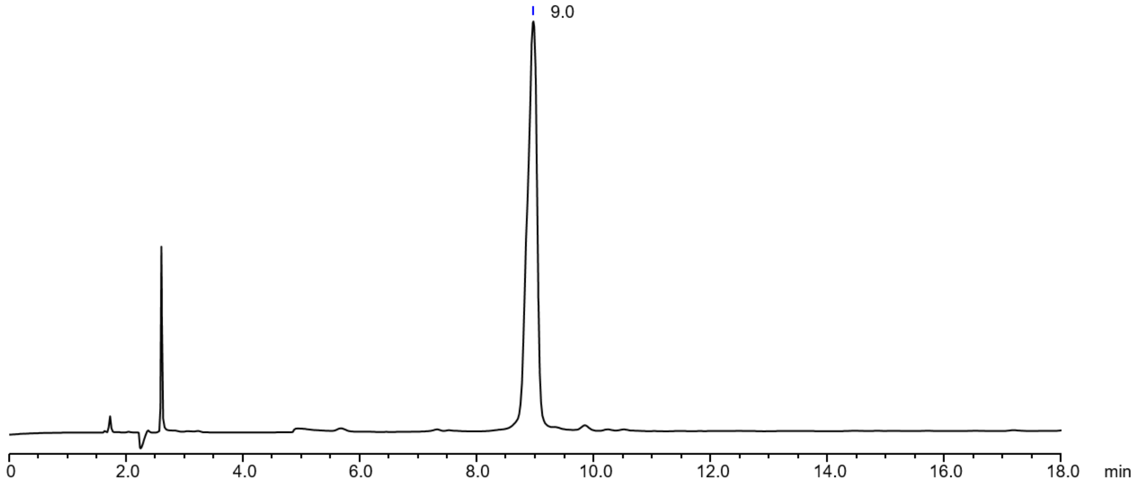

S1. RP-HPLC of *Fmoc-K(6-heptynoic acid)-GG-OH* at λ = 220 nm.

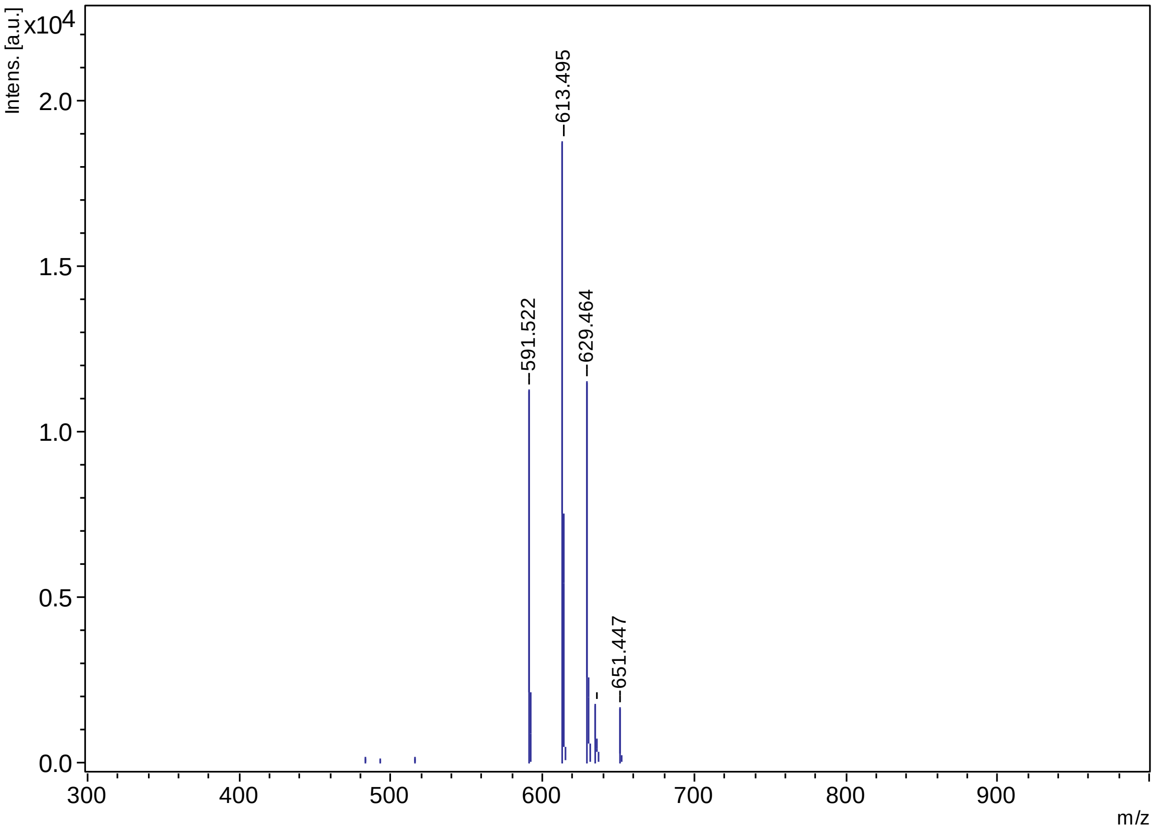

S2. Mass spectrum of *Fmoc-K(6-heptynoic acid)-GG-OH*.

2.2 NODAGA-TriLysan

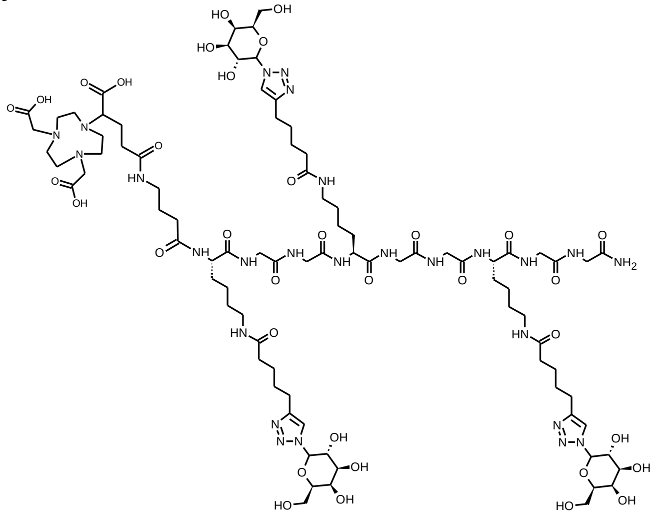

NODAGA-TriLysan

Mw: 2126.24 g/mol
Monoisotopic Mass: 2125.03 Da
$C_{88}H_{144}N_{26}O_{35}$

Fmoc-K(6-heptynoic acid)-GG-OH was loaded on to a *Rink amide* resin *(GP1),* followed by a Fmoc-deprotection step *(GP2).* Assembly of all building blocks was performed accordingly to *GP2b* and *GP3*. For coupling of Fmoc-protected γ-amino butyric acid (Fmoc-GABA) only 1.0 eq of HOAt, HATU and 4.5 eq of DIPEA were used. Cleavage of the unprotected peptide from the resin followed *GP6*.

For galactosylation the crude peptide (38 mg, 33 µmol, 1.0 eq) was dissolved in 100 µL H_2O/*tBu*OH (1:1) (vol/vol) and mixed with 1-Azido-1-deoxy-β-D-galactopyranoside tetraacetate (45 mg, 120 µmol, 3.6 eq) in 450 µL of hot H_2O/*tBu*OH/MeOH (1:1:1) (vol/vol/vol). Next, aqueous solutions of $Cu(OAc)_2$ (40.2 µmol, 1.2 eq) as well as sodium ascorbate (198 mg, 1 mmol, 30.0 eq) in a minimum amount of water were added. After 1 hour at 60 °C the galactosylated peptide was purified via semi-preparative HPLC (32-46 % B in 25 min) and lyophylized subsequently. For attachment of the chelator, 5 mg (3.2 µmol, 1.0 eq) of this intermediate were reacted with NODAGA-NHS (9 mg, 12.8 µmol, 4.0 eq) in 450 µL dry DMSO. The pH was adjusted to 8-9 with 22 µL (128 µmol, 40 eq) DIPEA. After 2 hours at room temperature the reaction was stopped by addition of 1 mL of Millipore water followed by removal of all volatiles *in vacuo.* For deacetylation the crude residue was treated over night with 3 mL of NEt_3/MeOH/H_2O (1:6:2). Final purification via semi-preparative HPLC (5-25 %B in 25 min) followed by lyophylization yielded 2.2 mg (1 µmol, 3 %) of a colorless solid.

Analytical HPLC (ReproSil Pur, 5-25 % B in 15 min, 1.0 mL/min) t_R = 11.0 min (18 % B).

MALDI-MS (m/z) = 2147.2 $[M+Na]^+$.

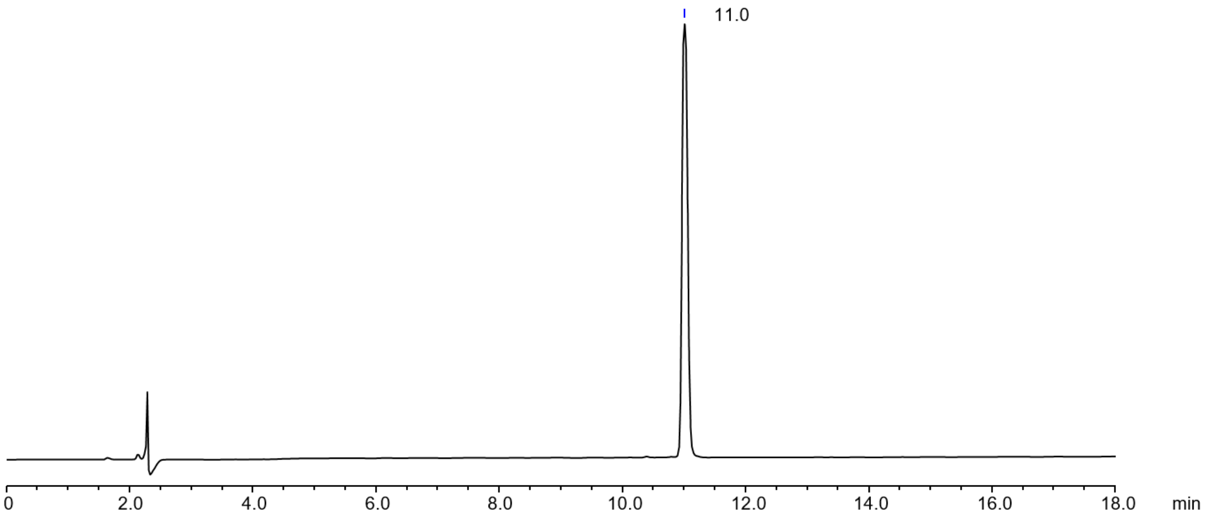

S3. RP-HPLC of *NODAGA-TriLysan* at λ = 220 nm.

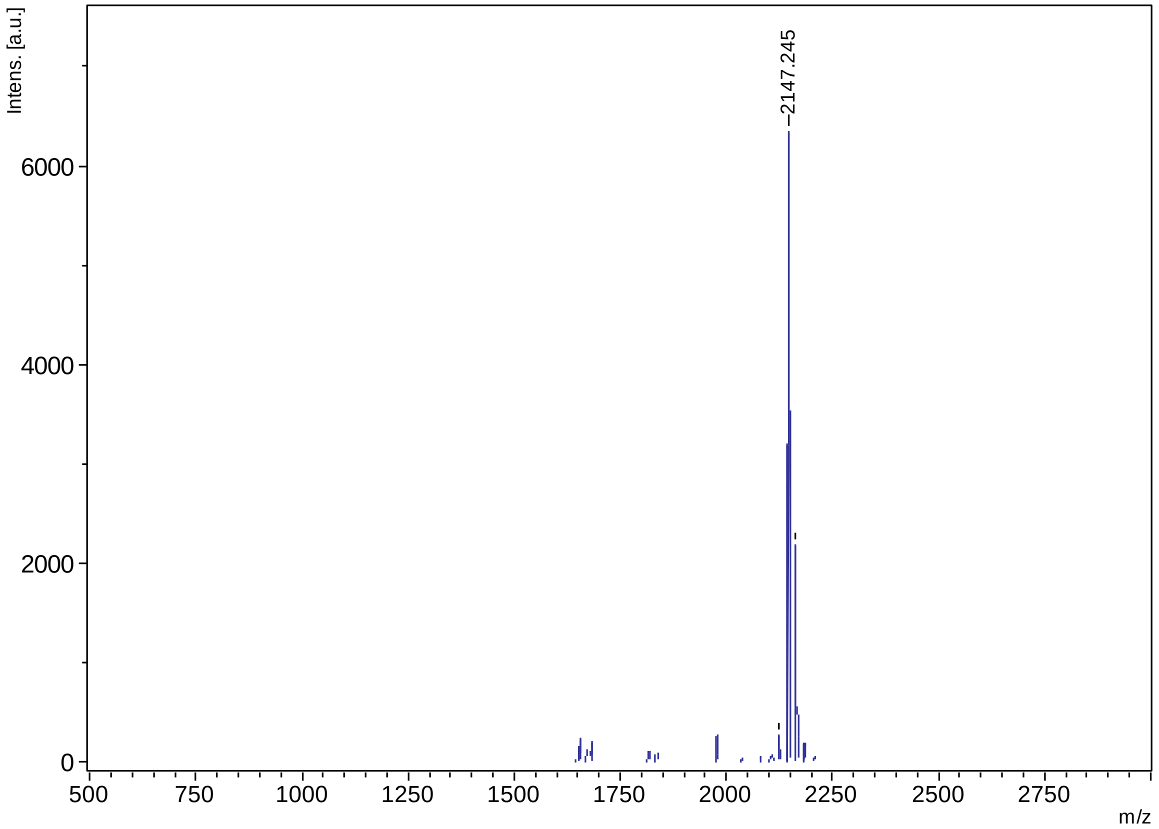

S4. Mass spectrum of *NODAGA-TriLysan.*

2.3 NODAGA-HexaLysan

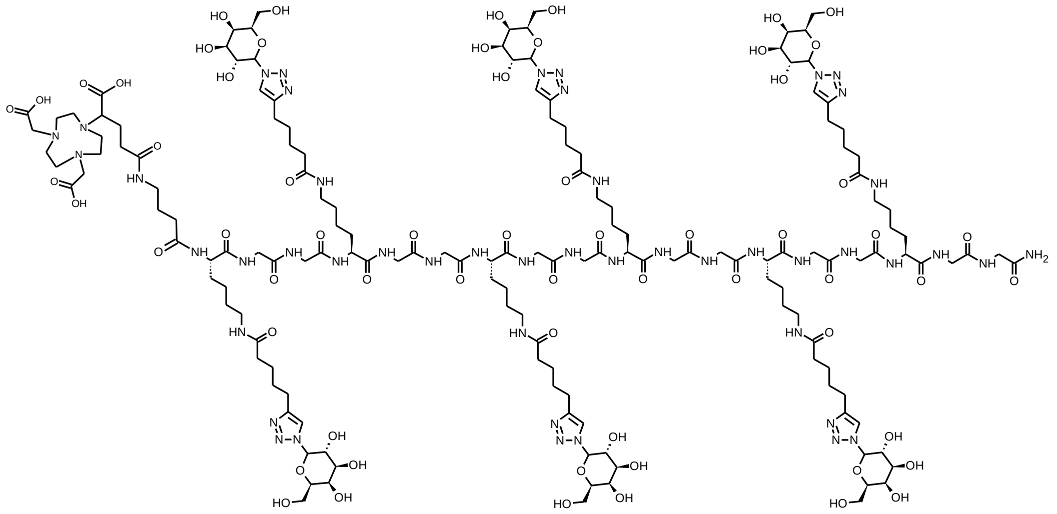

NODAGA-HexaLysan

Mw: 3792.98 g/mol
Monoisotopic Mass: 3790.82 Da
$C_{157}H_{255}N_{47}O_{62}$

Synthesis of the peptidic backbone followed the same principle as outlined for NODAGA-TriLysan with some exceptions. After each coupling a capping step was performed *(GP5)* to avoid formation of truncated peptide species. Subsequent to coupling of Fmoc-GABA (GP2b & GP3), the Fmoc-deprotected peptide was cleaved from the resin and directly lypohylized (GP6).

For galactosylation the crude peptide (44 mg, 20 µmol, 1.0 eq) was dissolved in 500 µL H_2O/*t*BuOH (1:1) and a solution of 1-Azido-1-deoxy-β-D-galactopyranoside tetraacetate (53.7 mg, 144 µmol, 7.2 eq) in hot H_2O/*t*BuOH/MeOH (1:1:1) (vol/vol/vol) was added. Next, solutions of $Cu(OAc)_2$ (4.8 mg, 24 µmol, 1.2 eq) and sodium ascorbate (158 mg, 800 µmol, 40.0 eq) in a small amount of water were added and the resulting mixture was incubated for 1 h at 60 °C. After centrifugation (3000 rpm, 5 min) the supernatant was removed, syringe filtered and the fully galactosylated peptide was isolated via semipreparative HPLC (43-55 % B in 25 min). For attachment of the chelator, 7.1 mg (1.5 µmol, 1.0 eq) of this intermediate were reacted with NODAGA-NHS (4.8 mg, 6.6 µmol, 4.3 eq) in 450 µL dry DMSO. The pH was adjusted to 8-9 with 10.5 µL (60 µmol, 40 eq) DIPEA. After 2 hours at room temperature the reaction was stopped by addition of 1 mL of Millipore water followed by removal of all volatiles *in vacuo.* For subsequent deacetylation the residue was treated over night with 3 mL of NEt_3/MeOH/H_2O (1:6:2) (vol/vol/vol). Removal of all volatiles and purification via semipreparative RP-HPLC (12-15 % B in 25 min) followed by lyophylization yielded 1.2 mg (316 nmol, 1 %) of a colorless solid.

Analytical HPLC (ReproSil Pur, 5-25 % B in 15 min, 1.0 mL/min) t_R = 11.8 min (19 % B).

ESI-MS (m/z) = 3793.82 $[M+H]^+$.

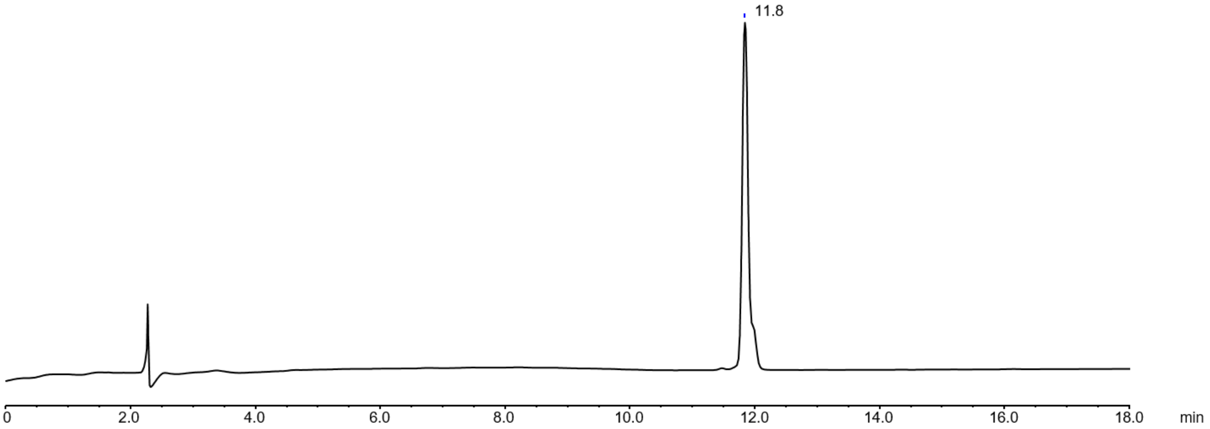

S5. RP-HPLC chromatogram of *NODAGA-HexaLysan* at λ = 220 nm.

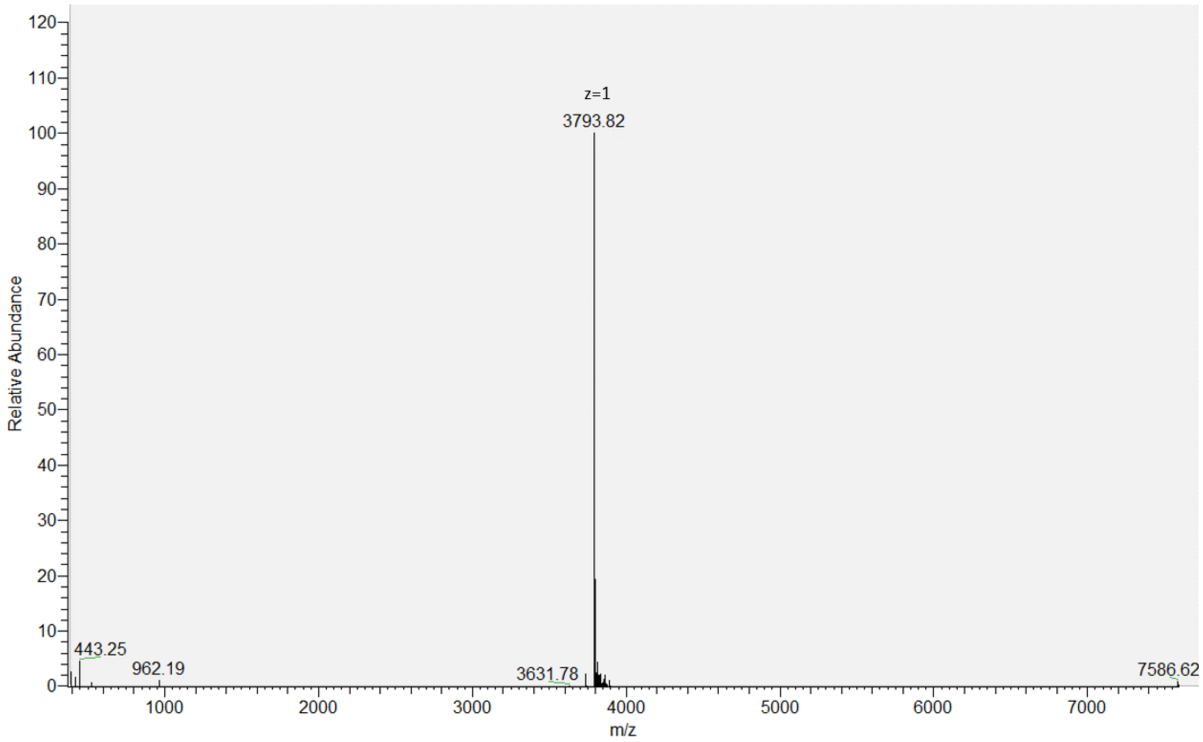

S6. Mass spectrum of *NODAGA-HexaLysan*.

2.4 NODAGA-NonaLysan

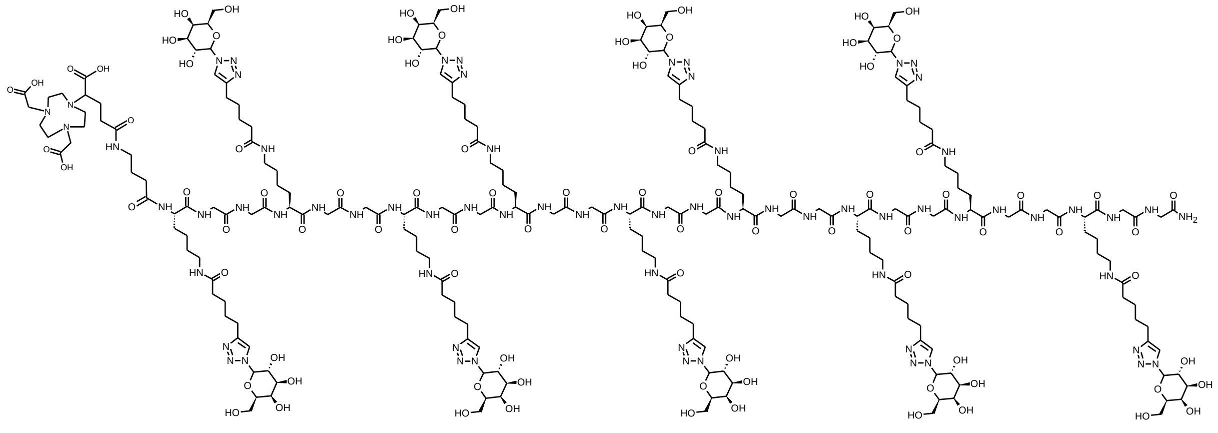

NODAGA-NonaLysan

Mw: 5459.73 g/mol
Monoisotopic Mass: 5456.62
$C_{226}H_{366}N_{68}O_{89}$

Synthesis of the peptidic backbone followed the same principle as outlined for NODAGA-TriLysan with some exceptions. After each coupling a capping step was performed *(GP5)* to avoid formation of truncated peptide species. Subsequent to coupling of Fmoc-GABA (GP2b & GP3), the Fmoc-deprotected peptide was cleaved from the resin and directly lypohylized (GP6).

For galactosylation the crude peptide (62 mg, 19 µmol, 1.0 eq) was dissolved in 500 µL H_2O/*t*BuOH (1:1) (vol/vol) and a solution of 1-Azido-1-deoxy-β-D-galactopyranoside tetraacetate (77.3 mg, 207 µmol, 10.8 eq) in hot H_2O/*t*BuOH/MeOH (1:1:1) (vol/vol/vol) was added. Next, solutions of $Cu(OAc)_2$ (4.6 mg, 23 µmol, 1.2 eq) and sodium ascorbate (153 mg, 768 µmol, 40.0 eq) in a small amount of water were added and the resulting mixture was incubated for 1 h at 60 °C. After centrifugation (3000 rpm, 5 min) the supernatant was removed, syringe filtered and the fully galactosylated peptide was isolated via semipreparative HPLC (43-55 % B in 25 min). For attachment of the chelator, 6.8 mg (1.0 µmol, 1.0 eq) of this intermediate were reacted with NODAGA-NHS (3 mg, 4.2 µmol, 4.2 eq) in 450 µL dry DMSO adjusted to pH 8-9 with 7 µL (40 µmol, 40 eq) DIPEA. After 2 hours at room temperature the reaction was stopped by addition of 300 µL of millipore water, followed by removal of all volatiles *in vacuo.* For subsequent deacetylation the residue was treated over night with 3 mL of NEt_3/MeOH/H_2O (1:6:2) (vol/vol/vol). All volatiles were removed *in vacuo* and the residue was purified via semipreparative HPLC (12-15 % B in 25 min). Subsequent lyophylization yielded 5 mg (916 nmol, 2 %) of a colorless solid.

Analytical HPLC (ReproSil Pur, 5-25 % B in 15 min, 1.0 mL/min) t_R = 12.3 min (20 % B).

ESI-MS (m/z) = 1820.88 $[M+3H]^{3+}$, 1365.91 $[M+4H]^{4+}$, 1092.93 $[M+5H]^{5+}$.

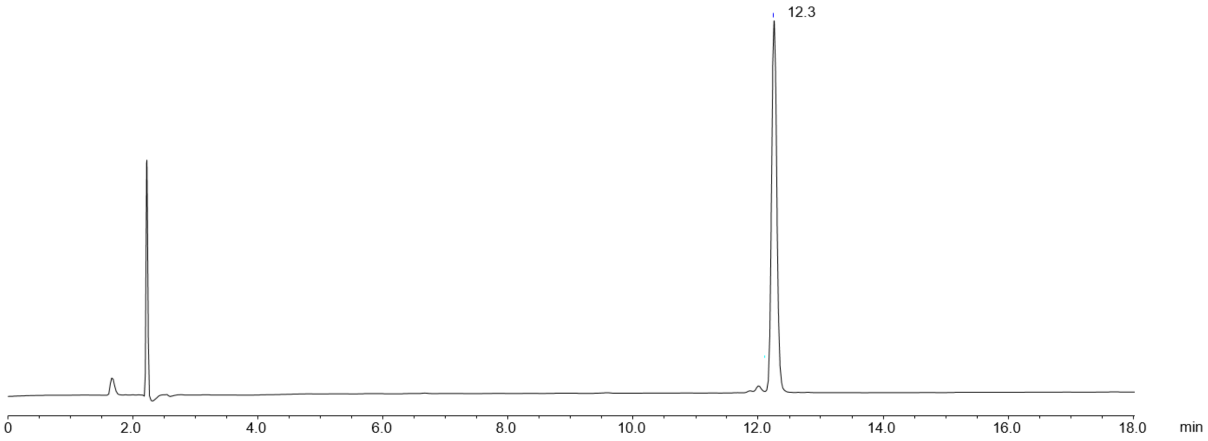

S7. RP-HPLC chromatogram of *NODAGA-NonaLysan* at λ = 220 nm.

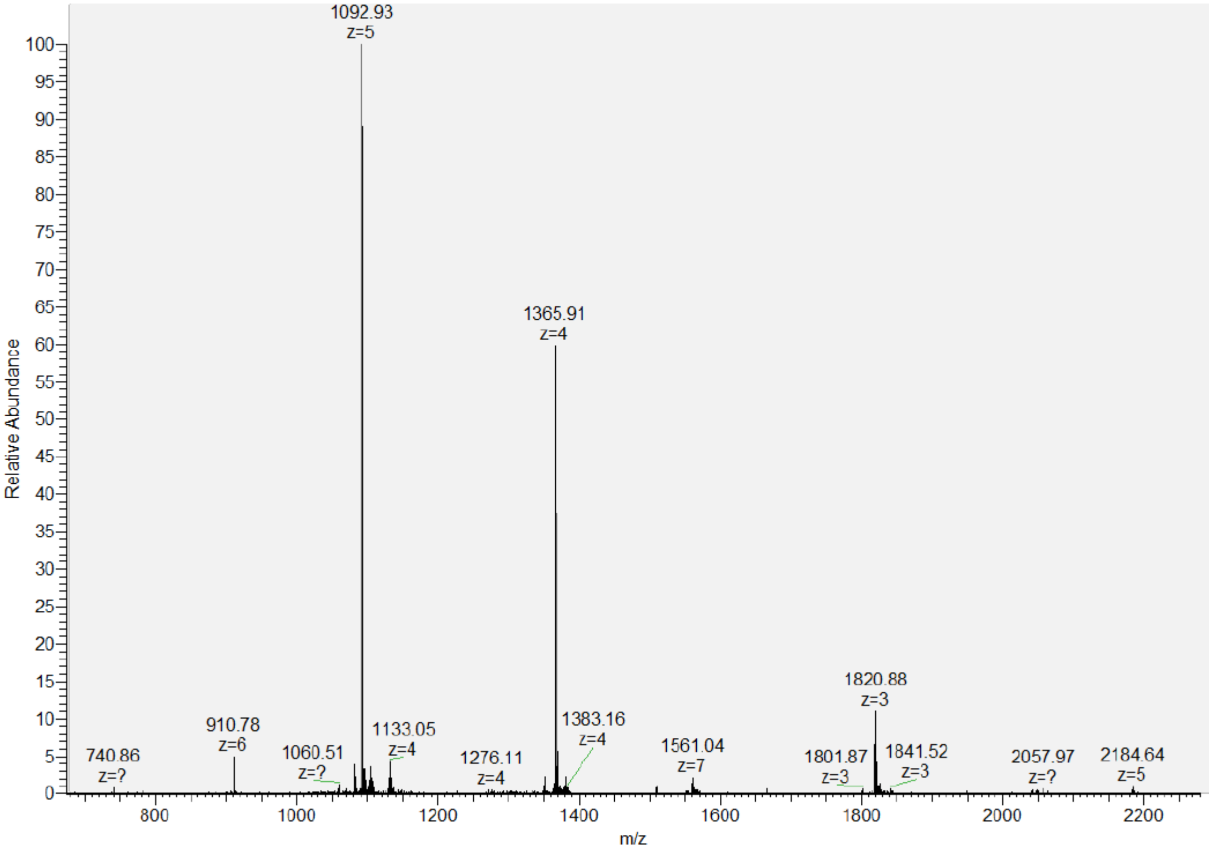

S8. Mass spectrum of *NODAGA-NonaLysan*.

2.5 *H-GalNAc-NonaLysan*

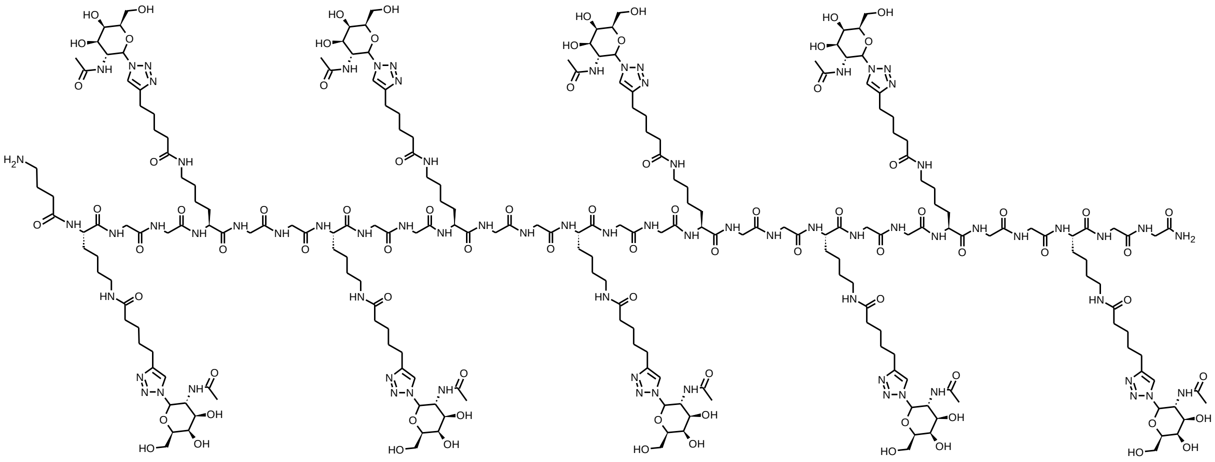

H-GalNAc-NonaLysan

Mw: 5471.83 g/mol
Monoisotopic Mass: 5451.70 Da
$C_{229}H_{370}N_{74}O_{82}$

Synthesis of the peptidic backbone followed the same principle as outlined for NODAGA-TriLysan with some exceptions. After each coupling a capping step was performed *(GP5)* to avoid formation of truncated peptide species. Subsequent to coupling of Fmoc-GABA (GP2b & GP3), the Fmoc-deprotected peptide was cleaved from the resin and directly lypohylized (GP6). For galactosylation the crude peptide (36 mg, 11 µmol, 1.0 eq) was dissolved in 400 µL H_2O/*t*BuOH (1:1) (vol/vol) and mixed with an aqueous solution of β-GalNAc-azide (25 mg, 101 µmol, 9.2 eq). Next, solutions of $Cu(OAc)_2$ (2.6 mg, 13.2 µmol, 1.2 eq) and sodium ascorbate (87 mg, 440 µmol, 40.0 eq) in a minimum amount of water were added. The mixture was incubated for1 hour at 60 °C and directly injected into semipreparative HPLC (12-15 % B in 25 min). Lyophylization yielded 7.6 mg (1.4 µmol, 13 %) of a colorless solid.

Analytical HPLC (ReproSil Pur, 5-25 % B in 15 min, 1.0 mL/min) t_R = 12.3 min (20 % B).

ESI-MS (m/z) = 5472.72 $[M+H]^+$.

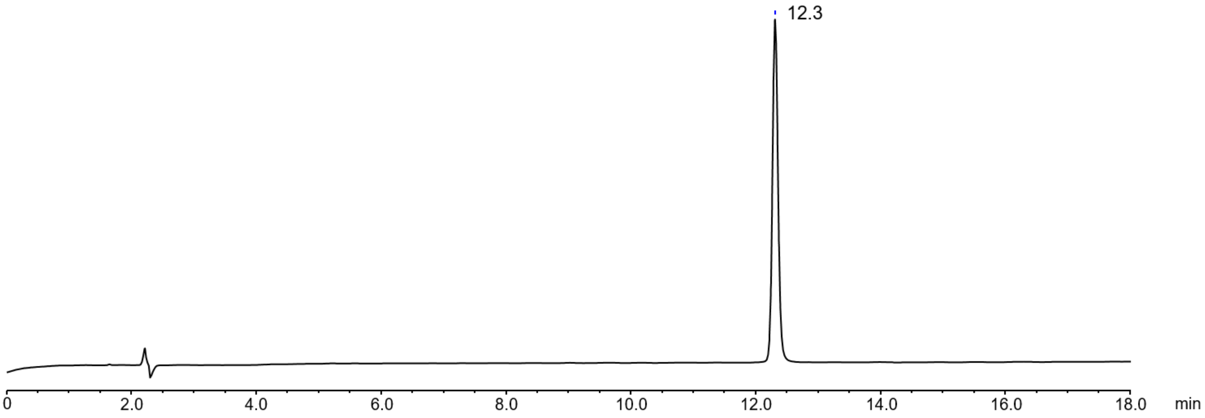

S9. RP-HPLC of *H-GalNAc-NonaLysan* at λ = 220 nm.

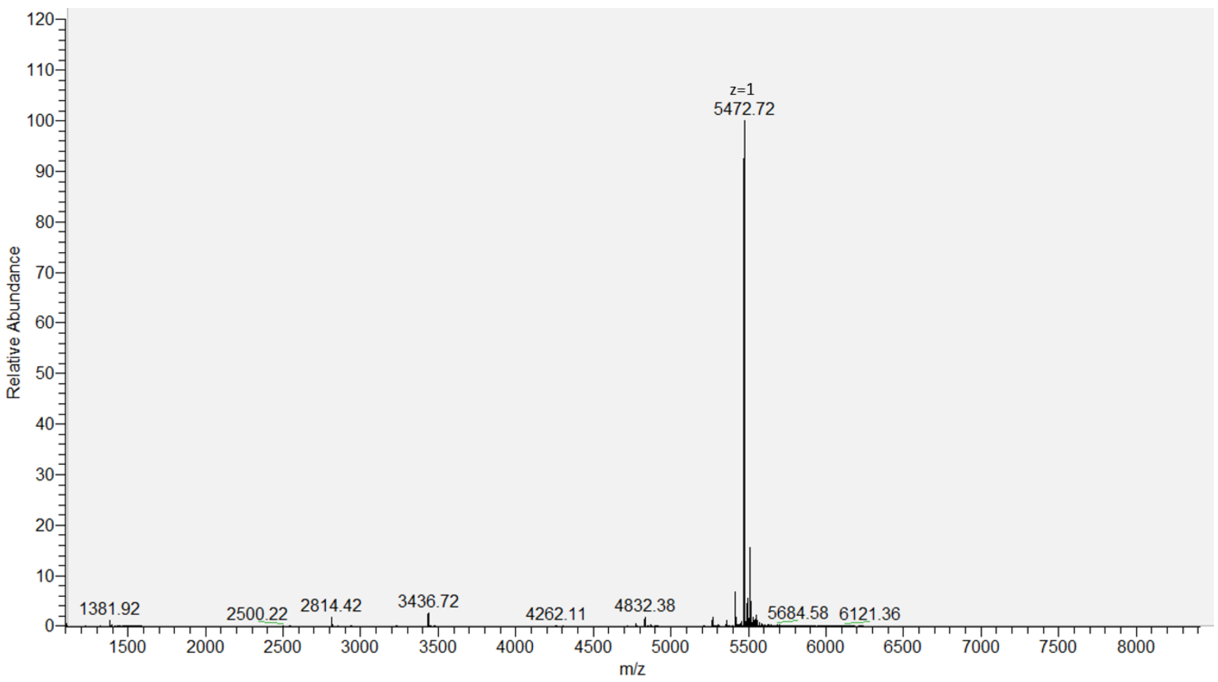

S10. Mass spectrum of *H-GalNAc-NonaLysan*.

2.6 NODAGA-GalNAc-NonaLysan

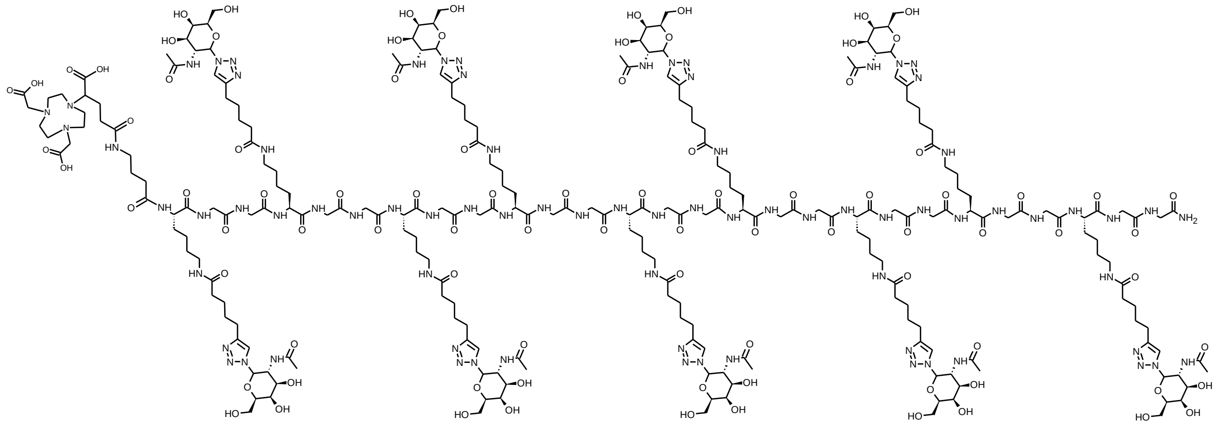

NODAGA-GalNAc-NonaLysan

Mw: 5829.19 g/mol
Monoisotopic Mass: 5825.86 Da
$C_{244}H_{393}N_{77}O_{89}$

H-GalNAc-NonaLysan (750 µg, 134 nmol, 1.0 eq) was reacted with NODAGA-NHS (400 µg, 548 nmol, 4.0 eq) in 90 µL of dry DMSO. The pH was adjusted to 8-9 with 0.5 µL (2.7 µmol, 20 eq) DIPEA. After 2 hours at room temperature the reaction mixture was diluted ten-fold with millipore water and directly subtracted to purification via analytical RP-HPLC (14 % B isocratic). Lyophylization of the product fraction yielded 250 µg (43 nmol, 32 %) of a colorless solid.

Analytical HPLC (ReproSil Pur, 5-25 % B in 15 min, 1.0 mL/min) t_R = 12.9 min (21 % B).

ESI-MS (m/z) = 5826.85 $[M+H]^+$.

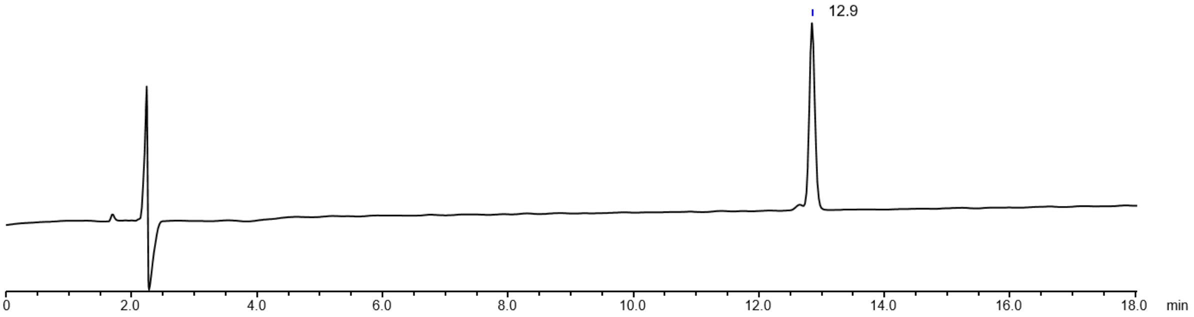

S11. RP-HPLC of *NODAGA-GalNAc-NonaLysan* at λ = 220 nm.

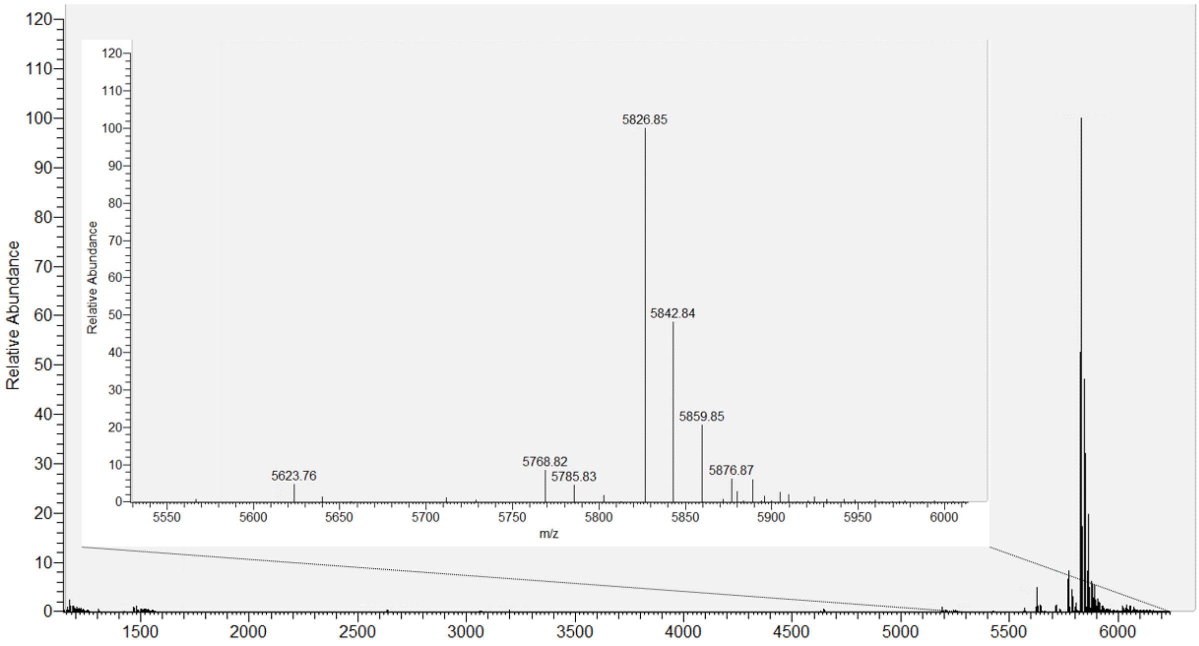

S12. Mass spectrum of *NODAGA-GalNAc-NonaLysan*.

3. Additional Tables

Table S1. Biodistribution of [^{68}Ga]Ga-**NODAGA-NonaLysan** (n=3) and [^{68}Ga]Ga-**NODAGA-HexaLysan** (n=3) in healthy BALB/c mice. Data are expressed as a percentage of the injected dose per gram (% ID/g), mean value ± standard deviation.

	[^{68}Ga]Ga-**NODAGA-NonaLysan**				[^{68}Ga]Ga-**NODAGA-HexaLysan**		
				**blocking**			
	10 min	30 min	60 min	30 min	10 min	30 min	60 min
**Liver**	71.2 ± 0.4	79.6 ± 8.0	69.6 ± 1.0	48.0 ± 1.1	54.8 ± 3.0	55.5 ± 7.4	45.9 ± 11.8
**Kidneys**	1.2 ± 0.1	0.8 ± 0.03	1.0 ± 0.2	2.0 ± 0.8	4.8 ± 0.3	1.9 ± 0.2	1.3 ± 0.3
**Blood**	0.4 ± 0.03	0.2 ± 0.04	0.2 ± 0.1	0.3 ± 0.2	2.1 ± 0.04	0.6 ± 0.1	1.1 ± 0.4
**Lung**	0.9 ± 0.1	0.7 ± 0.1	0.9 ± 0.1	0.6 ± 0.1	1.6 ± 0.2	0.8 ± 0.1	0.6 ± 0.2
**Femur**	0.3 ± 0.02	0.3 ± 0.1	0.2 ± 0.01	0.2 ± 0.01	0.5 ± 0.1	0.3 ± 0.03	0
**Heart**	0.4 ± 0.04	0.4 ± 0.04	0.5 ± 0.1	0.3 ± 0.03	0.9 ± 0.1	0.4 ± 0.1	0.3 ± 0.02
**Spleen**	0.6 ± 0.2	0.2 ± 0.03	0.5 ±0.03	0.3 ± 0.2	0.5 ± 0.1	0.2 ± 0.1	0.2 ± 0.02
**Intestine**	0.8 ± 0.2	5.6 ± 0.2	7.9 ± 0.5	2.2 ± 0.2	1.2 ± 0.3	4.0 ± 0.3	5.9 ± 1.7
**Stomach**	0.7 ± 0.2	1.2 ± 0.4	1.8 ± 1.0	0.9 ± 0.3	1.3 ± 0.3	1.2 ± 0.3	0.4 ± 0.1
**Pancreas**	0.8 ± 0.1	0.9 ± 0.1	1.2 ± 0.2	0.5 ± 0.1	0.9 ± 0.1	0.7 ± 0.1	0.5 ± 0.1
**Muscle**	0.6 ± 0.1	0.7 ± 0.03	0.6 ± 0.1	0.4 ± 0.04	0.9 ± 1.2	0.6 ± 0.03	0.4 ± 0.04

Table S2. Biodistribution of [^{68}Ga]Ga-**NODAGA-GalNAc-NonaLysan** (n=3) and [^{68}Ga]Ga-**NODAGA-TriLysan** (n=3) in healthy BALB/c mice. Data are expressed as a percentage of the injected dose per gram (% ID/g), mean value ± standard deviation.

	[^{68}Ga]Ga-**NODAGA-GalNAc-NonaLysan**				[^{68}Ga]Ga-**NODAGA-TriLysan**		
				**blocking**			
	10 min	30 min	60 min	30 min	10 min	30 min	60 min
**Liver**	69.4 ± 5.0	77.6 ± 8.0	57.6 ± 0.7	52.9 ± 3.2	9.5 ± 1.6	9.4 ± 2.0	9.2 ± 0.3
**Kidneys**	3.6 ± 2.7	1.5 ± 0.2	1.3 ± 0.02	1.9 ± 0.4	7.6 ± 1.4	5.0 ± 0.02	2.0 ± 0.3
**Blood**	0.9 ± 0.5	0.3 ± 0.1	0.3 ± 0.1	0.4 ± 0.1	3.6 ± 0.1	1.6 ± 0.2	0.3 ± 0.01
**Lung**	1.5 ± 0.1	1.5 ± 0.2	1.2 ± 0.04	1.3 ± 0.3	2.6 ± 0.2	1.2 ± 0.1	0.5 ± 0.1
**Femur**	0.9 ± 0.4	0.8 ± 0.1	0.9 ± 0.4	0.7 ± 0.3	0.9 ± 0.3	0.4 ± 0.02	0.1 ± 0.01
**Heart**	1.9 ± 0.5	1.7 ± 0.2	1.7 ± 0.1	0.8 ± 0.1	1.4 ± 0.01	0.6 ± 0.04	0.2 ± 0.01
**Spleen**	0.3 ± 0.1	0.3 ± 0.03	0.3 ± 0.1	0.3 ± 0.02	1.0 ± 0.01	0.4 ± 0.02	0.2± 0
**Intestine**	1.3 ± 0.3	3.5 ± 0.2	5.6 ± 0.03	1.9 ± 0.4	1.2 ± 0.3	1.1 ± 0.02	1.0 ± 0.3
**Stomach**	2.3 ± 0.4	2.7 ± 1.0	2.2 ± 0	2.4 ± 0.9	1.6 ± 0.2	0.9 ± 0.1	0.3 ± 0.01
**Pancreas**	5.0 ± 0.5	5.6 ± 0.6	5.0 ± 0.6	1.5 ± 0.8	1.0 ± 0.04	0.4 ± 0	0.2 ± 0.01
**Muscle**	1.3 ± 0.3	1.2 ± 0.1	1.1 ± 0.1	1.0 ± 0.3	0.9 ± 0.1	0.4 ± 0.04	0.1 ± 0
